# Integrated Metabolome, Transcriptome, and Physiological Analysis of the Flavonoid and Phenylethanol Glycosides Accumulation in Wild *Phlomoides rotata* Roots from Different Habitats

**DOI:** 10.3390/ijms26020668

**Published:** 2025-01-14

**Authors:** Zuxia Li, Guigong Geng, Chongxin Yin, Lianyu Zhou, Xiaozhuo Wu, Jianxia Ma, Rui Su, Zirui Wang, Feng Qiao, Huichun Xie

**Affiliations:** 1 Key Laboratory of Tibetan Plateau Medicinal Plant and Animal Resources, School of Life Sciences, Qinghai Normal University, Xining 810008, China; 18909789774@163.com (Z.L.); yinchongxin0701@163.com (C.Y.); 2025060@qhnu.edu.cn (L.Z.); xiaozhuo0623@163.com (X.W.); majianxia0926@163.com (J.M.); 18797079134@163.com (R.S.); wangzirui020124@163.com (Z.W.); 2 Academy of Agricultural and Forestry Sciences, Qinghai University, Xining 810016, China; 3 Qinghai South of Qilian Mountain Forest Ecosystem Observation and Research Station, Huzhu 810500, China; genggg-298@163.com; 4 Academy of Plateau Science and Sustainability, Qinghai Normal University, Xining 810008, China

**Keywords:** *Phlomoides rotata*, flavonoids, phenylethanol glycosides, metabolome, transcriptome, physiological index

## Abstract

*Phlomoides rotata*, a traditional medicinal plant, is commonly found on the Tibetan Plateau at altitudes of 3100–5200 m. Its primary active medicinal compounds, flavonoids and phenylethanol glycosides (PhGs), exhibit various pharmacological effects, including hemostatic, anti-inflammatory, antitumor, immunomodulatory, and antioxidant activities. This study analyzed flavonoid and PhG metabolites in the roots of *P. rotata* collected from Henan County (HN), Guoluo County (GL), Yushu County (YS), and Chengduo County (CD) in Qinghai Province. A total of differentially abundant metabolites (DAMs) including 38 flavonoids and 21 PhGs were identified. Six genes (*UFGT1*, *CHS1*, *COMT2*, *C4H3*, *C4H8*, and *C4H5*) and four enzymes (4CL, C4H, PPO, and ALDH) were found to play key roles in regulating flavonoid and PhG biosynthesis in *P. rotata* roots. With increasing altitude, the relative content of 15 metabolites, the expression of seven genes, and the activity of four enzymes associated with flavonoid and PhG metabolism increased. These findings enhance our understanding of the regulatory mechanisms of flavonoid and PhG metabolism in *P. rotata* and provide insights into the potential pharmaceutical applications of its bioactive compounds.

## 1. Introduction

Phenylpropanoid metabolism is a vital pathway in plants, generating over 8000 metabolites that significantly influence plant development and interactions with the environment [1]. Phenylpropanoid metabolism produces various metabolites, including lignin, flavonoids, lignans, phenylethanol glycosides (PhGs), hydroxycinnamic acid amides, and so on [2,3]. These compounds, derived from the phenylpropanoid pathway, feature a C6-C3-C6 benzene ring structure [4,5]. Flavonoids are essential for plant growth and development and have important applications in food and medicine. They have shown effectiveness in treating cancer, inflammatory disorders affecting the cardiovascular and nervous systems, and oxidative stress [4,5]. PhGs are a significant class of natural products derived from medicinal plants, numbering over 572 [6]. They possess various biological activities, including neuroprotective effects, anti-inflammatory properties [6], antioxidant activity [7], antibacterial effects [8], and antiviral activity [9]. The core structure of PhGs includes a hydroxyphenylethyl moiety attached to PhGs, typically featuring substituents, such as aromatic acids and various sugar esters or glycosidic bonds [10]. Verbascoside, the most well-known PhGs, contains a rhamnose portion, alongside hydroxysalidroside and a caffeoyl condensation residue, which contribute to its potential health benefits for humans [11]. Currently, PhGs have also been isolated from *Acanthus ilicifolius* [12], *Cistanche salsa* [13], *Cistanche deserticola* [14], and *Cistanche tubulosa* [15].

*Phlomoides rotata* is a perennial herb found in meadows, grasslands, and gravel areas at altitudes of 3100 to 5100 m [16]. *P. rotata* is primarily located in the Qinghai, Xizang, Sichuan, and Gansu Provinces of China, especially on the Qinghai–Tibet Plateau [17,18]. Research has focused on *P. rotata*’s traditional uses, chemical constituents, pharmacological effects, clinical applications, quality control, toxicology, and pharmacokinetics [19]. Approximately 127 chemical components, including flavonoids, PhGs, and iridoids, have been isolated from *P. rotata* [20]. These secondary metabolites exhibit various pharmacological properties, such as hemostatic [20,21,22], anti-inflammatory [23], antitumor [23], immunomodulatory [24], antioxidant [25], renal protective [26], and liver protective activities [27]. In total, at least 223 chemical constituents have been isolated from *P. rotata*, including PhGs, flavonoids, iridoids, and volatile oils [18]. Flavonoids are polyphenolic compounds widely present in the roots, stems, leaves, flowers, and fruits of plants [28,29]. More than 8000 flavonoids, such as anthocyanins, quercetin, catechins, apigenin, and luteolin, have been documented [30,31]. Studies indicate that dietary anthocyanins, flavonols, flavonoids, and isoflavones can reduce the risk of coronary heart disease and Alzheimer’s disease [32,33]. Forsythoside B, verbascoside, alyssonoside, isoverbascoside, and leucosceptoside B are among the PhGs isolated from *P. rotata* [34,35]. Notably, PhGs have been identified as the primary compounds responsible for its analgesic effects [36]. In Tibetan medicine, *P. rotata* is employed for treating “yellow water diseases”, which include skin conditions, jaundice, and rheumatism [37]. Our research examined 59 flavonoid metabolites and 29 differentially expressed genes (DEGs) related to the flavonoid pathway in *P. rotata* leaves from four different habitats in Qinghai [38]. Eleven flavonoids were found to increase with altitude, and the genes *PAL2*, *UFGT6*, *COMT1*, *HCT2*, *4CL4*, and *HCT3* were critical in regulating flavonoid biosynthesis [38].

This study conducted a comparative analysis of metabolomic and transcriptomic data in *P. rotata* roots, elucidating the expression patterns of genes involved in the flavonoid and PhG pathways. The findings contribute to a deeper understanding of the molecular mechanisms driving the biosynthesis of these compounds in *P. rotata*, thereby providing essential insights into the molecular regulatory mechanisms of flavonoid and PhG accumulation in the roots of *P. rotata*.

## 2. Results

### 2.1. Evaluation of the Metabolomics of P. rotata Roots from Four Different Habitats

**Figure 1:**
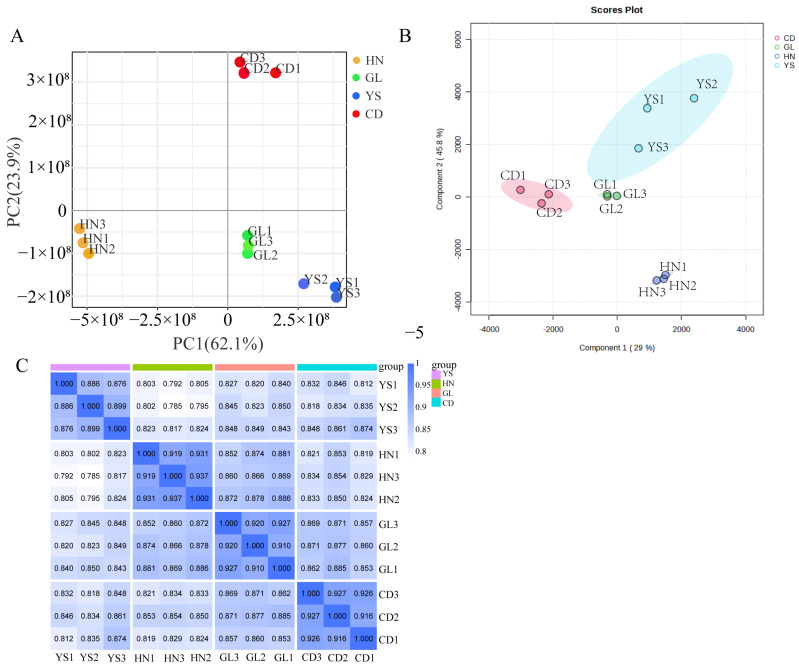
PCA, PLSD-DA, and SCA analysis of metabolites in *P. rotata* roots from four habitats. (**A**) The PCA analysis of *P. rotata roots* from four different habitats. (**B**) PLS-DA analysis of *P. rotata roots* from four different habitats. (**C**) SCA analysis of *P. rotata roots* from four different habitats.

Principal component analysis (PCA) is a statistical tool used to identify the internal structure among multiple variables by reducing them to a smaller number of principal components. The PCA results revealed that the explained variances for PC1 and PC2 were 62.1% and 23.9%, respectively (Figure 1A). This indicated that the three replicate samples within the same region were closely clustered; whereas, significant differences in metabolite distribution were observed across the four different habitats (Figure 1A). Specifically, samples from Chengduo County (CD) and Guoluo County (GL) were close along PC1 but exhibited substantial differences along PC2 (Figure 1A). In contrast, samples from Henan County (HN) and GL clustered closely along PC2; whereas, considerable diversity was observed along PC1 (Figure 1A).

The PLSD-DA model was also built for the purpose of classification and variable importance scoring (Figure 1B, Table S11). The significance of this model was assessed by a permutation test, and a p-value below 0.05 was obtained (Figure 1B). The VIP score for each variable was calculated to reveal the most contribution among intergroup differences in the PLSD-DA model (Figure 1B). 

Spearman’s correlation analysis (SCA) was performed on the metabolites of *P. rotata* roots collected from four distinct habitats to obtain reliable, high-quality metabolomic data. The SCA coefficients among the twelve samples of metabolites from four different habitats all exceeded 0.7, indicating reproducibility and reliability across the four habitats (Figure 1C).

Metabolomics analysis using LC-MS/MS was employed to identify both primary and secondary metabolites from the four habitats. Kyoto Encyclopedia of Genes and Genomes (KEGG) pathway analysis annotated the top 20 secondary metabolic pathways (Figure S1). The biosynthesis of flavonoids and PhGs in *P. rotata* roots from habitats was analyzed, identifying a total of five KEGG pathways related to flavonoid and PhG biosynthesis. A comparison of KEGG pathways revealed 18 pathways between HN and CD, 17 between GL and Yushu County (YS), and 15 between GL and CD (Table 1). Notably, both flavonoid biosynthesis and phenylalanine metabolism are part of the phenylpropanoid metabolism pathway.

### 2.2. Changes in the Content of Flavonoid and Phenylethanol Glycosides Metabolites in P. rotata Roots from Four Different Habitats

**Figure 2:**
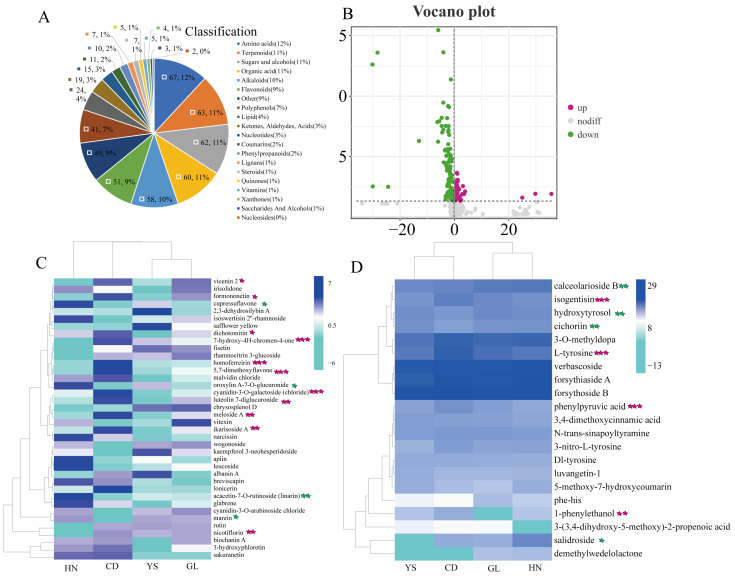
Metabolites profiling of *P. rotata* roots from four habitats. (**A**) Classification and proportion of all metabolites. (**B**) Volcano map of the distribution of metabolites among four habitats. The horizontal dash line means the boundary between significant and insignificant metabolites. (**C**) Heatmap of 38 flavonoid. (**D**) Heatmap of 21 PhGs. Light blue represents the low content, deep blue represents high content. Gray arrow represents a clustering branch in row and column. The five-pointed star represents the relationship between the content of metabolites and altitude. Green five-pointed star: negative relationship, red five-pointed star: positive relationship. One five-pointed star at *p* < 0.05, two five-pointed stars at *p* < 0.01, three five-pointed stars at *p* < 0.001.

To further assess the variations in metabolites among *P. rotata* roots across four distinct habitats, we employed the criteria of fold change (FC) > 1, *p* < 0.05, and VIP > 1 to identify differentially accumulated metabolites (DAMs). A total of 563 metabolites were detected (Table S1), which included 67 amino acids, 63 terpenoids, 60 organic acid, 51 flavonoids, 58 alkaloids, and 10 phenylpropanoids (Figure 2A). Among the metabolite categories, the proportions of flavonoids and phenylpropanoids were 9% and 2%, respectively (Figure 2A). 

A volcano plot illustrated the screening results of DAMs in *P. rotata* roots from four habitats (Figure 2B). Notably, 179 differential compounds were identified among four habitats, which comprised 122 downregulated and 57 upregulated compounds (Figure 2B). A total of 364 metabolites showed no difference (Figure 2B).

We identified 38 DAMs in the flavonoid metabolism pathway and 21 DAMs in the PhG metabolism pathway from *P. rotata* roots across four habitats (Table S2). A cluster heatmap analysis was conducted to assess variations in metabolite content, revealing specific distribution patterns of DAMs in the four habitats (Figure 2C,D). Among flavonoids, 9 compounds had elevated levels in the HN habitat, 14 in CD, 6 in YS, and 4 in GL (Figure 2C). In the PhG pathway, verbascoside, forsythiaside A, and forsythoside B exhibited high levels across all habitats. Additionally, two compounds showed high levels in HN, and four in both CD and GL (Figure 2C). As altitude increased, the content of 11 flavonoid types also increased (Figure 2C). These included vicenin 2 (*p* < 0.05), formononetin (*p* < 0.05), dichotomitin (*p* < 0.05), 7-hydroxy-4H-chromen-4-one (*p* < 0.001), homoferreirin (*p* < 0.001), 5,7-dimethoxyflavone (*p* < 0.001), cyanidin-3-O-galactoside (chloride) (*p* < 0.001), luteolin 7-diglucuronide (*p* < 0.01), meloside A (*p* < 0.01), ikarisoside A (*p* < 0.01), and nicotiflorin (*p* < 0.01). Conversely, the content of four flavonoid compounds, including cupressuflavone (*p* < 0.05), oroxylin A-7-O-glucuronide (*p* < 0.05), acacetin-7-O-rutinoside (linarin) (*p* < 0.01), and marein (*p* < 0.05), decreased (Figure 2C). 

Similarly, as the altitude increased, the content of four PhG compounds rose (Figure 2D). These included isogentisin (*p* < 0.001), L-tyrosine (*p* < 0.001), phenylpyruvic acid (*p* < 0.001), and 1-phenylethanol (*p* < 0.01). Additionally, the content of four other PhG compounds decreased, including calceolarioside B (*p* < 0.01), hydroxytyrosol (*p* < 0.01), cichoriin (*p* < 0.01), and salidroside (*p* < 0.05).

### 2.3. Transcriptomic Analysis of P. rotata Roots from Different Habitats

**Figure 3:**
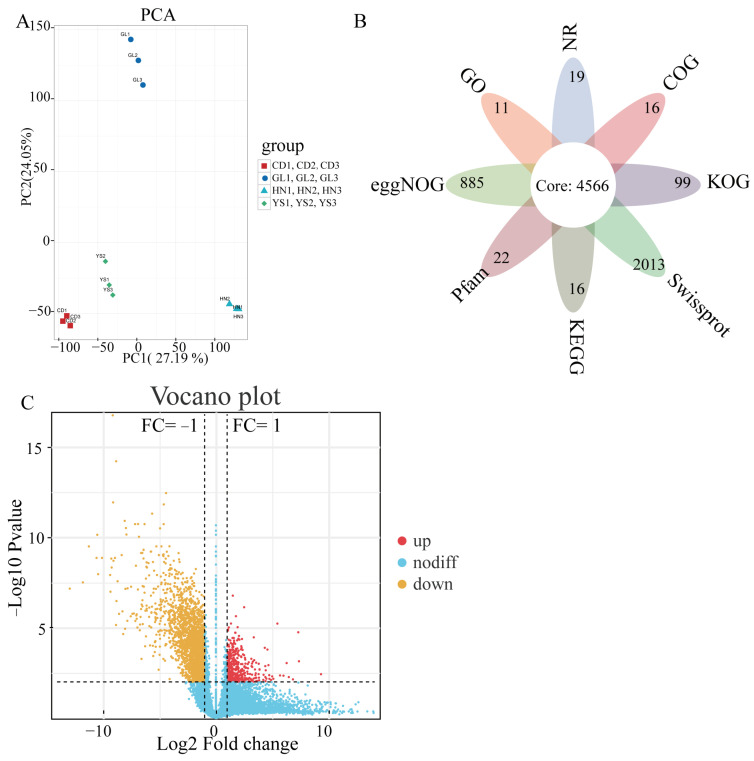
Transcriptomic analysis in *P. rotata* roots from four habitats. (**A**) Transcriptomic PCA of *P. rotata* roots from four different habitats. (**B**) Annotation of eight major databases. (**C**) Distribution of upregulation and downregulation of differentially expressed genes among four habitats. Upmodulated transcripts in red, downmodulated transcripts in yellow, nondifferent transcripts in blue. The horizontal dash line means the boundary between significant and insignificant of transcripts.

Transcriptome sequencing was conducted on 12 samples of *P. rotata* roots collected from four habitats. PCA results indicated that PC1 and PC2 explained 27.19% and 24.05% of the variance, respectively (Figure 3A). The analysis revealed that the three repetitions clustered closely; whereas, the four different regions were well-separated, highlighting significant differences in transcriptome profiles across these habitats (Figure 3A). In this study, the roots of *P. rotata* from four regions were used for transcriptome sequencing (Figure 3). The sequencing produced a total of 73.84 GB of data, with each sample providing at least 5.88 GB of clean data and a Q30 base percentage of 93.89% or higher (Table S3). The GC content varied from 44.55% to 46.03%, averaging 45.42% (Table S3). Trinity assembly was performed to eliminate adaptors, primer sequences, poly-A tails, and low-quality sequences (Figure S2). The final assembly dataset included 23,540 unique contigs, which were annotated using BLASTx across eight databases: NR, GO, eggNOG, Pfam, KEGG, Swiss-Prot, KOG, and COG (Figure 3B, Table S4). The KEGG database annotated 15,207 unigenes from the *P. rotata* roots across various habitats (Table S4). Among the eight major databases, 4566 shared unigenes were identified (Figure 3B).

To identify DEGs related to flavonoid and PhGs, we applied the criteria of FC > 2 and false discovery rate (FDR) < 0.01. DEGs were categorized into upregulated and down-regulated genes based on their relative expression levels (Tables S5 and S6). A comparison among four habitats revealed 4198 DEGs, consisting of 2702 downregulated and 1496 upregulated genes (Figure 3C, Table S7).

### 2.4. Key Genes Related to Flavonoid and Phenylethanoside Metabolism in P. rotata Roots from Different Habitats

**Figure 4:**
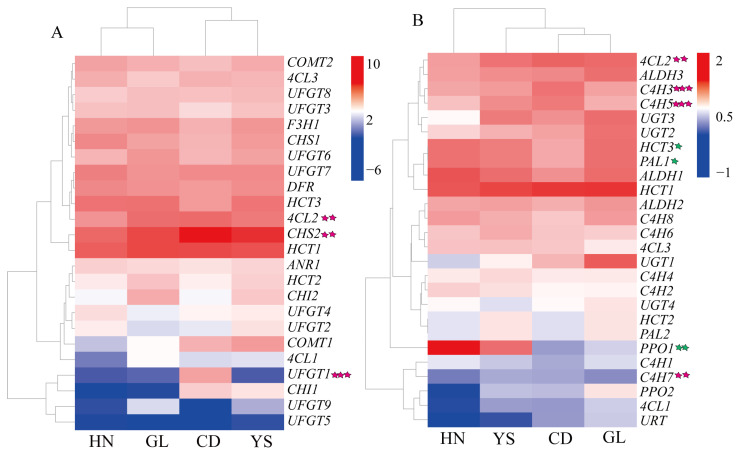
Heatmap of DEGs related flavonoid and phenylethanol glycoside pathways in *P. rotata* roots from four habitats. (**A**) Heatmap of DEGs related flavonoid pathway. (**B**) Heatmap of DEGs related phenylethanol glycoside pathway. Heatmap of all genes’ relative expression with Log10 FPKM. Red boxes indicate high expression levels, and blue boxes indicate low expression levels. The five-pointed star represents the relationship between gene expression and altitude. Green five-pointed star: negative relationship, red five-pointed star: positive relationship. One five-pointed star at *p* < 0.05, two five-pointed stars at *p* < 0.01, three five-pointed stars at *p* < 0.001. Gray arrow represents a clustering branch in row and column.

Based on KEGG pathway analysis and GO function analyses, this study identified 24 unigenes involved in flavonoid biosynthesis (Figure 4A, Table S5). These include nine *UFGT* genes, three *HCT* genes, three *4CL* genes, two *CHS* genes, two *CHI* genes, two *COMT* genes, one *DFR* gene, one *F3′H* gene, and one *ANR* gene (Figure 4A). Notably, *CHS2* and *HCT1* exhibited high expression across four habitats, while *UFGT5* and *UFGT9* demonstrated low expression (Figure 4A). As altitude increases, the expression levels of *4CL2*, *CHS2*, and *UFGT1* genes show a gradual increase (*p* < 0.01, *p* < 0.001).

Additionally, 26 unigenes involved in phenylethanol glycoside biosynthesis were identified. These include three *ALDH* genes, three *4CL* genes, eight *C4H* genes, three *HCT* genes, two *PAL* genes, two *PPO* genes, four *UGT* genes, and one *URT* gene (Figure 4B, Table S6). Overall, *HCT1* has high expression levels in all regions, while *PPO2*, *4CL1*, and *URT* exhibit lower expression levels in the HN regions. Conversely, *PPO1* shows high expression levels in the HN regions (Figure 4B). *HCT1* also demonstrates high expression across four habitats; whereas, *URT*, *4CL1*, and *C4H7* exhibit low expression (Figure 4B). As altitude increases, the expression of *4CL2*, *C4H3*, *C4H5*, and *C4H7* genes gradually increases (*p* < 0.01, *p* < 0.001), while the expression of *HCT3*, *PAL1*, and *PPO1* genes gradually decreases (*p* < 0.05, *p* < 0.01).

### 2.5. qRT-PCR Validation Among DEGs in Flavonoid and Phenylethanoside Metabolism

**Figure 5:**
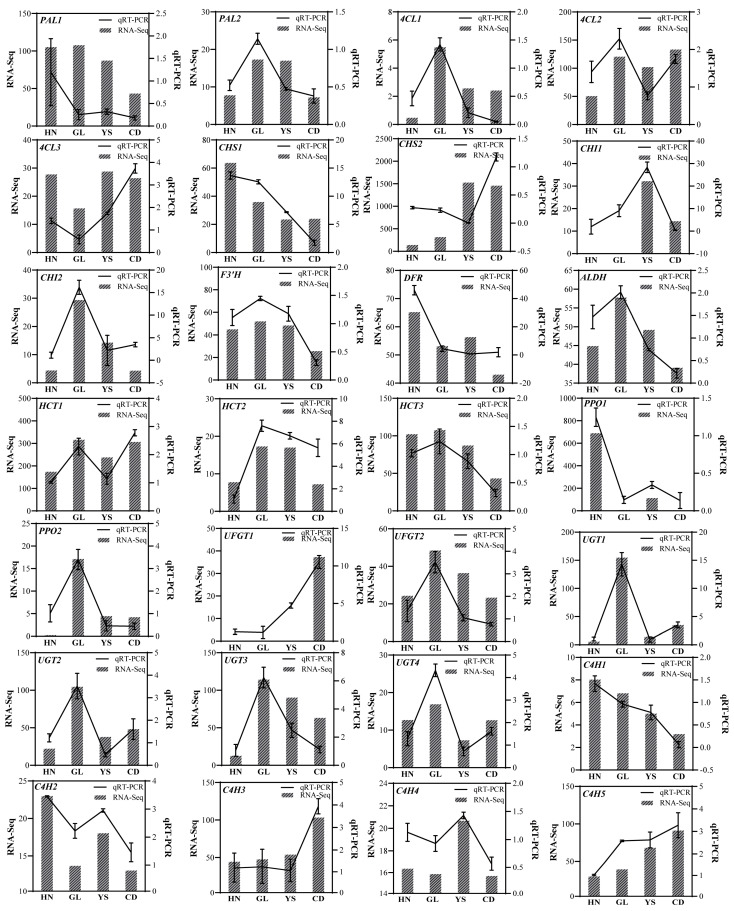
Validation of transcriptomic data by qRT-PCR analysis. The relative gene expression was calculated using the 2^−ΔΔct^ method. Vertical bars indicate means ± SD (3 replicates).

This study selected 28 genes involved in PhG and flavonoid biosynthesis to examine their expression patterns across four habitats using qRT-PCR. This process validated the transcriptomic analysis data (Figure 5). With the exception of the *PAL1* gene, the expression trends of the other genes assessed by qRT-PCR were consistent with the FPKM values from the transcriptomic analysis, reinforcing the reliability of the transcriptomic data (Figure 5). The relative expression levels of the *PAL1*, *CHS1*, *DFR*, and *C4H1* genes demonstrated a decreasing trend (Figure 5). Conversely, the relative expression of the *CHS2*, *UFGT1*, *C4H3*, and *C4H5* genes showed an increasing trend (Figure 5). The expression patterns of the *PAL2*, *4CL1*, *CHI1*, *CHI2*, *F3′H*, *ALDH*, *HCT2*, *HCT3*, *PPO2*, *UFGT2,* and *UGT3* genes initially increased before subsequently decreasing (Figure 5). However, the expression of the genes *4CL2*, *HCT1*, *UGT1*, *UGT2*, *UGT4*, *C4H2*, and *C4H4* exhibited irregular trends (Figure 5).

### 2.6. Changes in Enzyme Activity in Flavonoid and Phenylethanol Glycosides Biosynthesis in P. rotata Roots from Four Habitats

**Figure 6:**
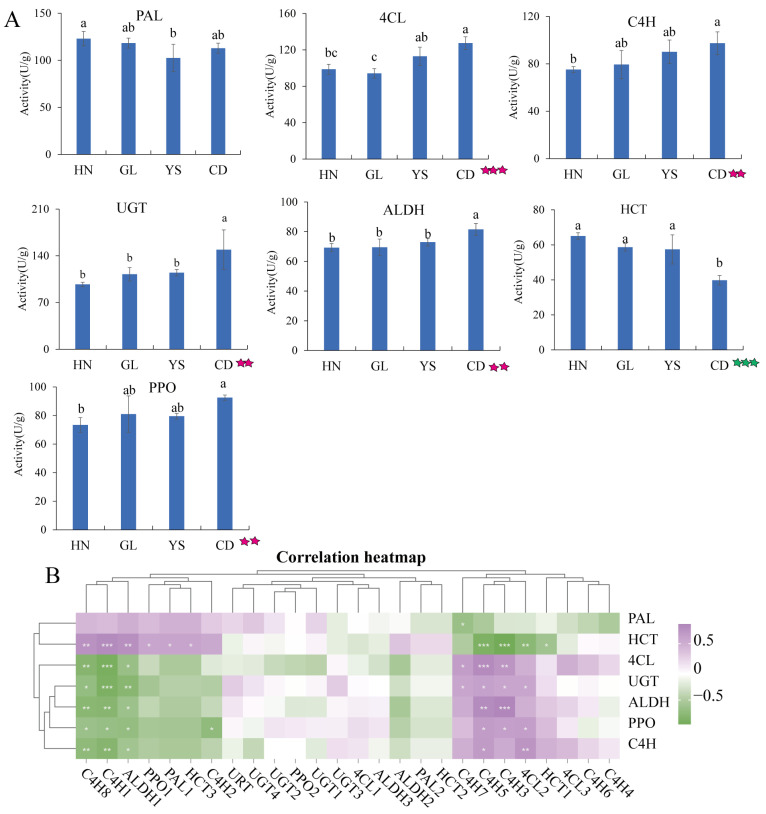
Enzyme activities related to flavonoid and PhG metabolism. (**A**) Seven enzymes activities. The five-pointed star represents the relationship between the enzyme activity and altitude. Green five-pointed star: negative relationship, red five-pointed star: positive relationship. Two five-pointed stars at *p* < 0.01, three five-pointed stars at *p* < 0.001. (**B**) Correlation analysis between gene expression and enzyme activity. Gray arrow represents a clustering branch in row and column. One star at *p* < 0.05, two stars at *p* < 0.01, three stars at *p* < 0.001.

Enzyme activity was evaluated using the enzyme-associated immunosorbent assay (ELISA) to quantify the activity of seven enzymes. The activities of 4CL, C4H, UGT, ALDH, and PPO showed an increasing trend; whereas, HCT displayed a decreasing trend (Figure 6A). High levels of PAL activity were observed in HN; whereas, low levels were recorded in YS (Figure 6A). As altitude increases, the activities of enzymes 4CL, C4H, UGT, ALDH, and PPO rise (*p* < 0.01, *p* < 0.001); whereas, that of the HCT enzyme declines (*p* < 0.001).

A positive correlation was found between the HCT enzyme activity and gene *HCT3* expression, as well as between enzyme C4H activity and gene *C4H5* expression (*p* < 0.05, Figure 6B). In contrast, a negative correlation was noted between enzyme HCT activity and gene *HCT1* expression (*p* < 0.05, Figure 6B), as well as between enzyme C4H activity and gene *C4H1*/*C4H8* expression (*p* < 0.01, Figure 6B).

### 2.7. Correlation Analysis Between the Level of Flavonoid and Phenylethanol Glycosides Metabolites and Expression of Key Genes

**Figure 7:**
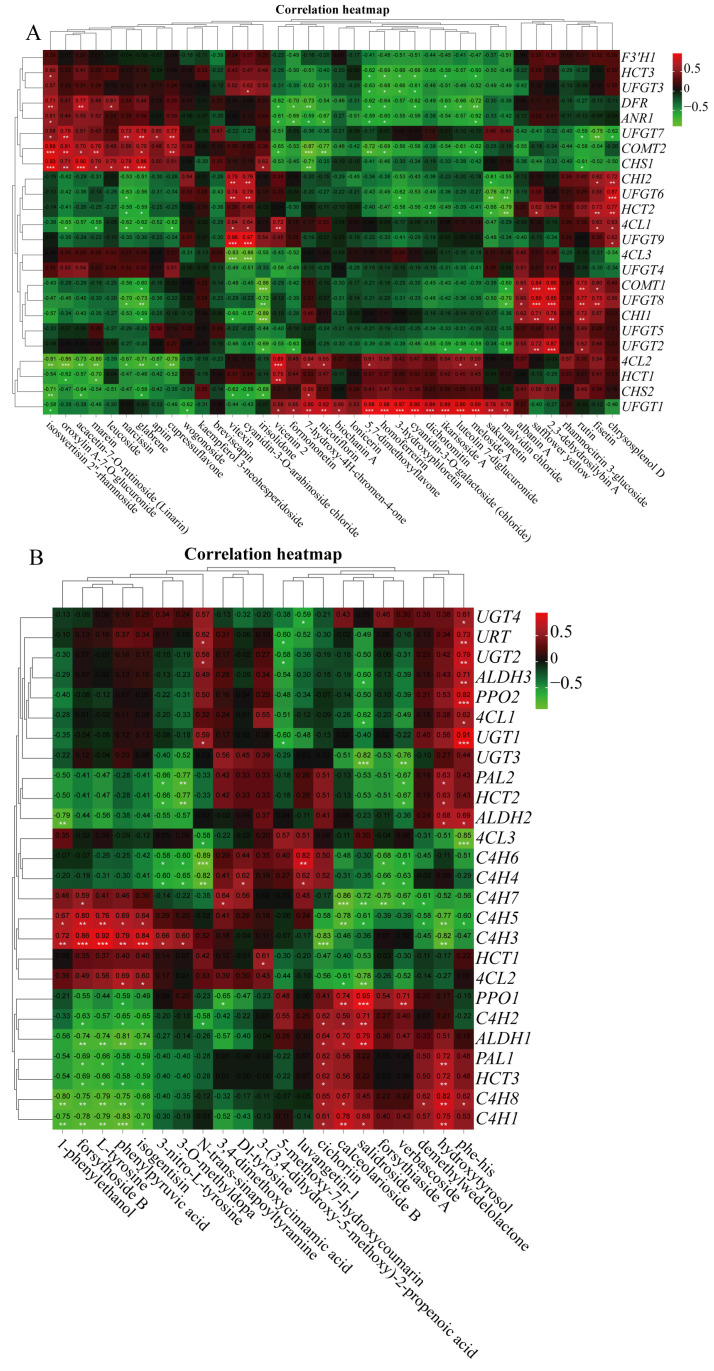
Correlation between genes expression and the content of the major metabolites in *P. rotata* roots from four habitats. (**A**) Correlation between genes expression and the content of the flavonoid compounds. (**B**) Correlation between genes expression and the content of the phenylethanoid glycoside compounds. Gray arrow represents a clustering branch in row and column. One star at *p* < 0.05, two stars at *p* < 0.01, three stars at *p* < 0.001.

Correlation analysis was performed to investigate the association between 38 flavonoid metabolite levels and the expression of 24 DEGs in the flavonoid biosynthesis pathway, using Pearson’s correlation analysis (Figure 7A). The analysis revealed 82 positive and 86 negative significant correlations (Figure 7A, *p* < 0.05, *p* < 0.01, *p* < 0.001). The expressions of *COMT2* and *CHS1* showed positive correlations with four compounds: isoswertisin 2″-rhamnoside, oroxylin A-7-O-glucuronide, acacetin-7-O-rutinoside (Linarin), and marein. Conversely, the expression of *4CL2* exhibited negative correlations with these compounds (Figure 7A). Additionally, the expression of *COMT1*, *UFGT8*, and *CHI1* demonstrated positive correlations with four compounds: albanin A, safflower yellow, 2,3-dehydrosilybin A, and rutin (Figure 7A). Furthermore, *UFGT1* exhibited positive correlations with 15 compounds, including 5,7-dimethoxyflavone, homoferreirin, and 3-hydroxyphloretin (Figure 7A). These findings suggested that *UFGT1* and *CHS* were key genes in the regulation of flavonoid biosynthesis.

Correlation analysis was conducted between the levels of 21 PhG metabolites and the expression levels of 26 DEGs involved in the PhG biosynthesis pathway, using Pearson’s correlation analysis (Figure 7B). The analysis identified 57 positive correlations and 69 negative correlations with statistical significance (Figure 7B, *p* < 0.05, *p* < 0.01, *p* < 0.001). The expression levels of *C4H5* and *C4H3* were positively correlated with five compounds: 1-phenylethanol, forsythoside B, L-tyrosine, phenylpyruvic acid, and isogentisin. Conversely, *C4H8* and *C4H1* showed negative correlations with these compounds (Figure 7B). Additionally, *UGT4, URT, UGT2, ALDH3, PPO2, 4CL1, UGT1, ALDH2*, and *C4H8* exhibited positive correlations with phe-his (Figure 7B). This suggested that *C4H3* played a key role in regulating PhG biosynthesis.

### 2.8. Correlation Analysis Between the Level of Flavonoid and Phenylethanoside Metabolites and the Activities of Key Enzymes

**Figure 8:**
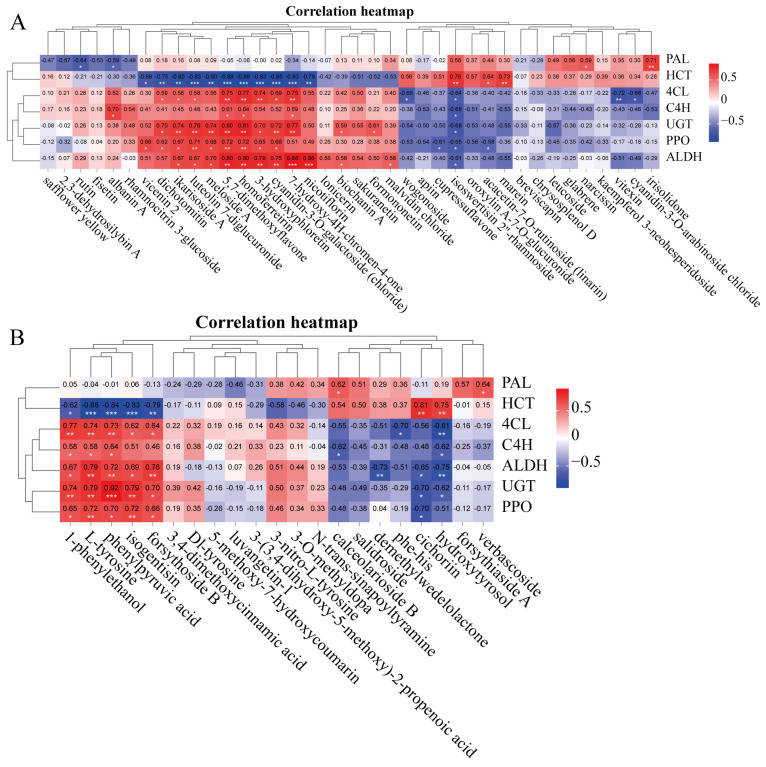
Correlation between key enzyme activity and major metabolite in *P. rotata* roots from four habitats. (**A**) Correlation between key enzyme activity and the content of the flavonoid compound. (**B**) Correlation between key enzyme activity and the content of the phenylethanoid glycoside compound. Gray arrow represents a clustering branch in row and column. One star at *p* < 0.05, two stars at *p* < 0.01, three stars at *p* < 0.001. Gray arrow represents a clustering tree, clustering by rows and columns.

A combined analysis of 38 flavonoid metabolites and seven key enzymes was performed using Pearson’s correlation analysis (Figure 8A). This analysis revealed 47 positive correlations and 23 negative correlations between flavonoids and key enzymes (Figure 8A, *p* < 0.05, *p* < 0.01, *p* < 0.001). The activities of 4CL, UGT, PPO, and ALDH were clustered together and showed significant positive correlations with seven compounds: ikarisoside A, luteolin 7-diglucuronide, meloside A, 5,7-dimethoxyflavone, 3-hydroxyphloretin, homoferreirin, and cyanidin-3-O-galactoside (chloride). In contrast, the activity of HCT was significantly negatively correlated with these compounds (Figure 8A).

Additionally, a combined analysis of 21 PhG metabolites and seven key enzymes was conducted using Pearson’s correlation analysis (Figure 8B, *p* < 0.05, *p* < 0.01, *p* < 0.001). This analysis showed 27 positive correlations and 15 negative correlations between PhG and the key enzymes. The activities of 4CL, ALDH, UGT, and PPO were clustered together, demonstrating significant positive correlations with five PhG compounds: 1-phenylethanol, L-tyrosine, phenylpyruvic acid, isogentisin, and forsythoside B. In contrast, HCT activity was clustered with significant negative correlations to these compounds content (Figure 8B). These findings highlight the vital roles of ALDH and UGT in regulating the biosynthesis of flavonoids and PhGs.

## 3. Discussion

### 3.1. Key Genes and Metabolites in Flavonoids and Phenylethanol Glycosides Metabolism from P. rotata Roots

Key enzyme genes, such as *HCT*, *CCR*, *COMT*, *CHS*, *F3H*, and *FLS*, positively regulate the metabolites involved in flower development in *Prunus mume* [39]. In contrast, *PAL*, *C4H*, and *4CL* negatively regulate these metabolites [39]. A total of 364 flavonoid metabolites from the roots, stems, leaves, and flowers of *Hemerocallis citrina* were identified, showing significant variations in the metabolic profiles among these plant parts [40]. The most abundant flavonoid metabolites were flavonols and flavones, followed by flavanones, chalcones, flavanols, flavanonols, anthocyanidins, tannins, and proanthocyanidins [40]. Shi et al. studied the accumulation of flavonoids in the fruit peel of purple and yellow passion fruit (*Passiflora edulis*) and analyzed the expression of related genes [41]. Genes *C4H*, *4CL*, *UFGT*, and *GST* may regulate flavonoid metabolism in the passion fruit peel [41]. Lei et al. annotated 19 differential flavonoid metabolites and 34 DEGs associated with the flavonoid metabolic network [42]. They identified eight key candidate genes involved in flavonoid metabolism [42]. The *ANS* gene was crucial for synthesizing cyanidin-3-O-glucoside; whereas, the *CHI*, *F3′H*, and *FLS* genes mainly controlled the levels of flavanones, flavones, and flavonols, respectively [42]. Yu et al. investigated *UFGT* activity, expression levels, and the accumulation of flavonoid glycosides in mulberry leaves [43]. The results demonstrated a strong positive correlation between the accumulation of isoquercitrin and astragalin and *UFGT* gene expression and activity [43].

In this study, 38 DAMs and 24 DEGs related to flavonoid synthesis were identified. Additionally, 21 DAMs and 26 DEGs associated with PhG synthesis were found. The results showed that 50 genes had significant positive or negative correlations with the levels of most flavonoids or PhGs in *P. rotata* (Figure 7). Specifically, the expressions of *UFGT1*, *CHS1*, and *COMT2* were clustered and positively correlated with 16, 8, and 6 flavonoids, respectively (Figure 7, *p* < 0.05, *p* < 0.01, *p* < 0.001). Similarly, *C4H3*, *C4H8*, and *CH41* were clustered and positively correlated with seven, five, and four PhGs, respectively (Figure 7, *p* < 0.05, *p* < 0.01, *p* < 0.001). Therefore, six genes—*UFGT1*, *CHS1*, *COMT2*, *C4H3*, *C4H8*, and *CH41*—are crucial for regulating flavonoid biosynthesis and PhGs. These findings offer valuable insights into the regulatory mechanisms of flavonoids and PhG biosynthesis, establishing a solid foundation for identifying key genes in future research.

A total of nine flavonoid compounds in the leaves of *P. rotata* were analyzed [38]. Among these, kaempferol 3-neohesperidoside, sakuranetin, and biochanin A exhibited high levels in HN [38]. Limocitrin and isoquercetin were elevated in YS, while ikarisoside A and chrysosplenol D were notably high in GL [38]. Schaftoside, miquelianin, malvidin chloride, and glabrene showed increased levels in CD [38]. The content levels of five flavonoids—luteolin, apiin, kaempferol 3-neohesperidoside, lonicin, and luteolin—were higher in *P. rotata* leaves than in *P. rotata* roots. Conversely, cyanidin-3-O-galactoside (chloride), luteolin 7-diglucuronide, vitexin, and ikarisoside A exhibited relatively high expression levels in both the roots and leaves (Figure S3A). The analysis of the remaining 21 flavonoids showed low content in the roots but higher content in the leaves (Figure S3B). 

### 3.2. Key Enzyme in Flavonoids and Phenylethanolic Glycosides Metabolism from P. rotata Roots

The characteristics of enzymes involved in plant specialized metabolism, such as flavonoid biosynthesis, are often marked by promiscuity [44]. A recent study identified key enzyme-encoding genes in *Cannabis sativa* that are crucial for flavonoid biosynthesis [45]. Notably, the *CsFLS* genes are responsible for producing flavonols, contributing to the diverse functions of flavonoid production in *C. sativa* [45]. Correlation analyses showed a significant positive association between the flavonoid content in *Tartary buckwheat* sprouts and the specific activities of key enzymes (PAL, CHI, FLS) (*p* < 0.01) [46]. This activity was also dynamically correlated with gene expression (*FtPAL*, *FtCHI*, *FtFLS1*, *FtFLS2*) related to their synthesis [46]. Glycosyltransferases, a large family of enzymes, are essential for covalently associating sugar monosaccharides to various organic substrates [47]. They play a vital role in synthesizing complex oligosaccharides called glycans, which are crucial for intercellular interactions across all life kingdoms [47]. These enzymes also catalyze sugar attachment during the formation of small-molecule metabolites, such as plant flavonoids [47]. The *Cyclocarya paliurus* GT1 protein was heterologously expressed in *Escherichia coli* and exhibited catalytic activity towards multiple flavonoids, favoring specific biosynthesis pathways [48]. *UFGT1* effectively catalyzed the glucosylation of flavones and flavonols; although, its activity was lower toward isoflavones, flavanones, or triterpenes in *C. paliurus* [49]. The *UGT* family, the largest glycosyltransferase superfamily in plants, primarily glycosylates hormones and phenylpropanoids using UDP-sugar as a donor [49].

**Figure 9:**
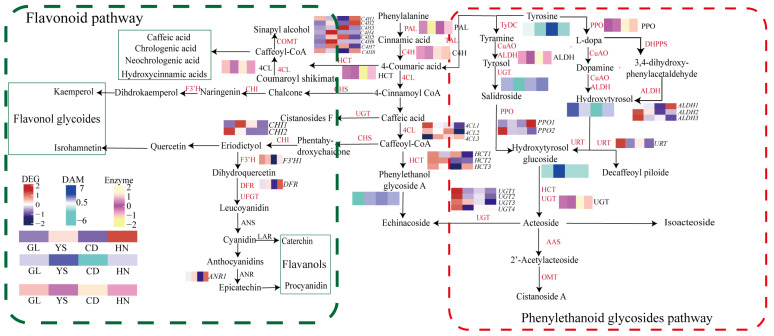
Metabolic pathways of flavonoids and phenylethanoid glycosides in plants. The box diagram in green represents the flavonoid pathway. The box diagram in red represents the phenylethanoid glycoside pathway. In the heatmap, the transcript expressions of 28 genes are in green font, activities of seven enzymes are in light blue, and the contents of five metabolites are in dark purple.

In our study, the combined analysis of enzymes and metabolites revealed a positive correlation between the activity of UGT or ALDH and the relative content of 11 flavonoids or five PhGs (Figure 8, *p* < 0.05, *p* < 0.01, *p* < 0.001). Additionally, PPO activity was positively correlated with the content of nine flavonoids and five PhGs (Figure 8, *p* < 0.05, *p* < 0.01, *p* < 0.001). Furthermore, 4CL activity showed a positive correlation with the content of eight flavonoids or five PhGs (Figure 8, *p* < 0.05, *p* < 0.01, *p* < 0.001). These findings indicate that the four enzymes—4CL, UGT, PPO, and ALDH—are crucial in regulating the biosynthesis of flavonoids and PhGs in the roots of *P. rotata* (Figure 9).

### 3.3. Effect of Altitude on Flavonoid and Phenylethanolic Glycoside Biosynthesis

Altitude significantly influences the accumulation of flavonoids in plants. Specifically, higher altitudes result in an increase in flavonoid content across various species [50]. Du et al. examined the leaves of *C. paliurus* at low altitude (280 m) and high altitude (920 m) using metabolomic and transcriptomic analyses [50]. Their findings showed that the flavonoid content and composition were greater in leaves of *C. paliurus* collected at higher altitudes compared to those from lower altitudes [50]. They identified 31 flavonoids that were significantly correlated with 227 DEGs, resulting in 412 related pairs (283 positive and 129 negative correlations). The variation in flavonoid accumulation at different altitudes may be attributed to differences in the transport and relocation of flavonoids within the leaves of *C. paliurus*, rather than differing biosynthesis pathways [50]. In another study, the chlorogenic acid (CGA) content in Huaxingyangyu and Jianchuanhong peaked at an altitude of 2800 m, followed by a slight decrease at 3300 m [51]. A total of 20 CGAs and intermediate compounds were identified, including 3-o-caffeoylquinic acid, 4-o-caffeoylquinic acid, and 5-o-caffeoylquinic acid [51]. Liu et al. conducted an integrated metabolomic and transcriptomic study to explore how cultivation at various altitudes—low (800 m), moderate (1800 m), and high (3600 m)—affects flavonoid biosynthesis in pigmented potato tubers [51]. The results indicated that both red and purple potato tubers grown at high altitudes had the highest flavonoid content and pigmentation, followed by those grown at low altitudes [51]. Co-expression network analysis revealed three modules of genes positively correlated with altitude-responsive flavonoid accumulation. Notably, the anthocyanin repressors *StMYBATV* and *StMYB3* showed a significant positive association with flavonoid accumulation related to altitude [52].

A combined analysis of genes and metabolites at various altitudes was conducted. Our results showed that the expression levels of *CHS2*, *UFGT1*, *4CL2*, *C4H3*, *C4H5*, and *C4H7* were highest in high-altitude areas. Correspondingly, the levels of 11 flavonoid compounds—including vitexin, formononetin, dichotomitin, 7-hydroxy-4H-chromen-4-one, homoferreirin, 5,7-dimethoxyflavone, cyanidin-3-O-galactoside (chloride), luteolin 7-diglucuronide, meloside A, ikarisoside A, and nicotiflorin—along with four PhG compounds (isogentisin, L-tyrosine, phenylpyruvic acid, and 1-phenylethanol) increased (Figure 2C,D). In contrast, the highest expression levels of *PAL1*, *PAL2*, *HCT1*, and *4CL2* genes were found in low-altitude areas, where the content of four flavonoid compounds (cupressuflavone, oroxylin A-7-O-glucuronide, acacetin-7-O-rutinoside (linarin), and marein) and four PhG compounds (calceolarioside B, hydroxytyrosol, cichoriin, and salidroside) also increased (Figure 7). Additionally, the activities of 4CL, UGT, ALDH, and PPO enzymes were highest in high-altitude areas (Figure 6A). Altitude is a significant factor contributing to the accumulation of flavonoids and PhGs. The differential accumulation of flavonoids and PhG metabolites across different altitudes may be attributed to changes in the transport and relocation of flavonoids within the roots of *P. rotata*. Furthermore, the upregulation of genes associated with energy and protein synthesis may enhance flavonoid accumulation in high-altitude regions.

## 4. Materials and Methods

### 4.1. Plant Materials

**Figure 10:**
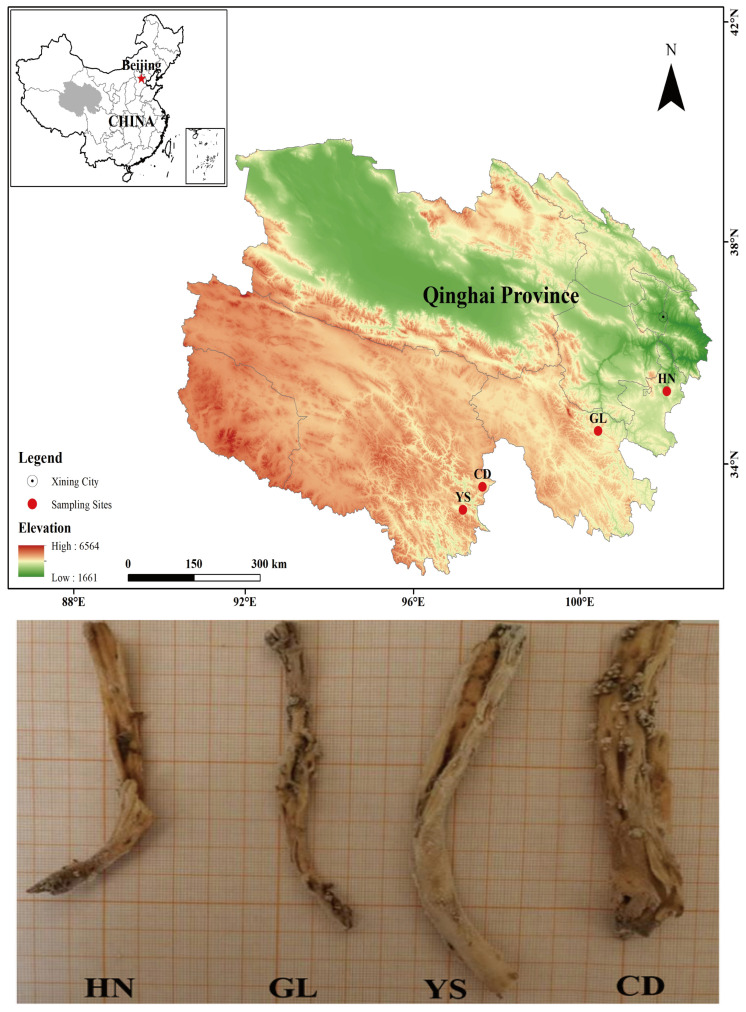
The roots of *P. rotata* from HN, GL, YS, and CD habitats.

Seedlings of *P. rotata* were collected in four different habitats in Qinghai Province (Figure 10, Table 2). Including Henan County (HN) at an altitude of 3540 m, Guoluo County (GL) at an altitude of 3750 m, Yushu County (YS) at an altitude of 3880 m, and Chengdu County (CD) at an altitude of 4270 m. In this study, 10–15 healthy plants with stable growth were randomly selected from each region and washed with water multiple times to remove topsoil. Subsequently, the plants were disinfected with 75% ethanol for 5 min, rinsed twice with sterile water, and immediately frozen in liquid nitrogen. The frozen samples were stored in a −80 °C freezer for subsequent metabolomic and transcriptomic analysis.

### 4.2. Extraction of Metabolites and LC-MS/MS Conditions

Metabolites were extracted from the roots of *P. rotata* in different regions with four biological replicates. Samples were vacuum freeze-dried, weighed, added with extraction solution, ground, sonicated, and stored at −80 °C. Then, they were centrifuged, the supernatant was collected, dried, dissolved with acetonitrile solution, mixed, centrifuged again, and the final supernatant was collected and stored for LC-MS/MS detection [38]. LC-MS/MS analysis was carried out using a specific ultrahigh-performance liquid chromatography tandem mass spectrometer. The conditions for ultrahigh-performance liquid chromatography and mass spectrometry parameters were set as described (with details in relevant columns and Table S8).

### 4.3. Qualitative Analysis of Metabolites and Principal Component Analysis

Metabolite mass spectrometry data of the different samples were obtained by Analyst 1.6.3. We integrated the peak areas of all mass spectral peaks, calculated relative content by peak area normalization method. We identified compounds’ classification and pathway info based on self-built GB-PLANT database. We calculated VIP values and *p* values by *t*-test and screened DAMs with FC > 1, *p* < 0.05 and VIP > 1. The PCA of metabolites was performed using prcomp (R base function).

Metabolite quantification was achieved via multiple reaction monitoring (MRM) analysis with triple quadrupole mass spectrometry. In the MRM mode, the four stages of rod screen precursor ions of the target substance eliminated interference, then the precursor ions were ionized and broken into fragment ions, which were filtered to select characteristic fragment ions for more accurate quantification. After obtaining metabolite mass spectrometry data from different samples, peak area integration and integration correction for the same metabolite’s peaks in different samples were carried out [53].

### 4.4. Transcriptome Sequencing and Data Analyses

Total RNA from different regions of *P. rotata* roots was extracted using TRIzol® reagent (Invitrogen, St. Louis, MO, USA). RNA concentration, purity, and integrity were assessed by (Thermo Scientific, St. Louis, MO, USA) and Agilent 2100 (Agilent Technologies Inc., SantaClara, CA, USA). An amount of 1 μg of RNA per group was used to construct a sequencing library with the NEBNext® Ultra™ RNA Library Prep Kit for Illumina® (NEB, Ipswich, MA, USA). Double-stranded cDNA fragments were size-selected by the AMPure XP system and then PCR-enriched to form a cDNA library. The Illumina NovaSeq 6000 sequencing platform was used for PE150 mode sequencing. Sequencing data were analyzed on the BMKCloud online platform. Raw data were processed by in-house perl scripts to obtain clean data, and relevant quality metrics (Q20, Q30, GC-content, sequence duplication level) were calculated. Downstream analyses were based on high-quality clean data. Trinity software was used to assemble the clean data from *P. rotata* with specific assembly parameters set (Figure S4B).

All assembled transcripts were searched via BLASTX against NCBI NR, COG, and KEGG databases (with E-value < 1.0 × 10^−5^) to obtain functional annotations. Trinity sequence assembly was carried out after obtaining high-quality sequencing data [54]. We used DIAMOND (v 2.0.4) [55] software to align the unigene sequence with the NR [56], Swiss Prot [57], COG [58], KOG [59], eggNOG4.5 [60], and KEGG [61] databases. Differentially expressed genes were analyzed by GO and KEGG pathway enrichment analyses. Gene expression levels were assessed by RSEM with FPKM for quantification. Differential expression analyses were screened using specific criteria with DESeq2 [62]. KEGG enrichment analysis was conducted by KOBAS [63]. Unique gene sequences related to flavonoid and PhG metabolism were identified from *P. rotata’s* transcriptome sequences in four regions. Online PCA was performed across the four regions. Gene-specific primers were designed by Online Primer Premier software (version 5.0) by Aoke DingshengBiotechnology Co., Ltd. (Xian, China). The primers used are listed in Table S9. RT–qPCR was carried out with specific reagents and a set reaction procedure (Table S10), with negative control and replicates, on a QuantStudio 6 Flex PCR instrument and analyzed by the 2^−△△Ct^ method [64].

### 4.5. Determination of Key Enzyme Activity in Flavonoids and Phenylethanol Glycosides Biosynthesis Pathway

In the flavonoids and PhG biosynthesis pathway, we selected seven key enzymes (PAL, 4CL, UGT, C4H, HCT, PPO, and ALDH) for activity determination using ELISA kit, and the enzyme activity assay kit in plant was provided by Jiangsu Boshen Biotechnology Co., Ltd. (Nanjing, China). The enzyme activity assay kit numbers are shown in Table 3.

### 4.6. Correlation Analysis of Metabolites and Key Genes

Correlation analysis was performed on metabolites and genes related to the biosynthesis pathways of flavonoids and PhGs. Additionally, we analyzed the correlation between key enzymes and metabolites, as well as the association between metabolites and altitude within these metabolic pathways. All correlation analysis heat maps with tags were generated using the OmicShare tool (https://www.omicshare.cn, accessed on 10 October 2024), with one star indicating *p* < 0.05, two stars indicating *p* < 0.01, and three stars indicating *p* < 0.001.

## 5. Conclusions

This study employed widely used untargeted metabolomics technology to assess the compositional characteristics of flavonoid and PhG metabolites in *P. rotata* roots from four distinct habitats. KEGG enrichment analysis revealed a relationship between most flavonoid metabolites and the biosynthesis pathways of phenylpropanoids, flavonoids, and secondary metabolites. A total of 38 flavonoids and 21 PhGs were identified in *P. rotata* roots. Among these, 11 flavonoids (vitexin, formononetin, dichotomitin, 7-hydroxy-4H-chromen-4-one, homoferreirin, 5,7-dimethoxyflavone, cyanidin-3-O-galactoside (chloride), luteolin 7-diglucuronide, meloside A, ikarisoside A, and nicotiflorin) and four PhGs (isogentisin, L-tyrosine, phenylpyruvic acid, and 1-phenylethanol) exhibited significant differences. The findings highlight six key genes—*UFGT1*, *CHS1*, *COMT2*, *C4H3*, *C4H8*, and *C4H5*—that are vital in regulating flavonoid and PhG biosynthesis in *P. rotata* roots. Furthermore, five enzymes (4CL, C4H, UGT, ALDH, and PPO) are essential for regulating the metabolism of these compounds. This study represents the first systematic analysis of flavonoid and PhG metabolites across the various habitats of *P. rotata* roots, laying a scientific foundation for future research in these environments.

## Figures and Tables

**Table 1:** The numbers of flavonoid and phenylethanol glycoside biosynthesis pathway in *P*. *rotata* roots between two habitats.

ko_ID	Metabolic Pathway	Groups of Two Habitats					
		HN vs. CD	GL vs. YS	GL vs. CD	HN vs. GL	HN vs. YS	YS vs. CD
ko00941	Flavonoid biosynthesis	3	3	4	3	3	4
ko00944	Flavone and flavonol biosynthesis	5	4	4	4	4	4
ko00943	Isoflavonoid biosynthesis	1	0	0	0	0	1
ko00360	Phenylalanine metabolism	5	6	3	4	0	5
ko00940	Phenylpropanoid biosynthesis	4	4	4	3	3	0
Total		18	17	15	14	10	14

**Table 2:** *Phlomoides rotata* samples from 4 habitats.

Sample	Location	Altitude	East Longitude	North Latitude
HN	Henan County, Qinghai Province	3540 m	101°56′48′	34°76′12″
GL	Guoluo County, Qinghai Province	3750 m	100°14′38′′	34°29′10″
YS	Yushu County, Qinghai Province	3880 m	97°1′23′′	32°51′4″
CD	Chengduo County, Qinghai Province	4270 m	97°27′16′′	33°18′2″

**Table 3:** The enzyme activity assay kit numbers in flavonoids and PhG biosynthesis pathway.

ELISA Kit	Batch Number	Product Number
PAL	20231124053 T	BS-E17999O2
4CL	20231124054 T	BS-E17986O2
UGT	20231211017 T	BS-E19655O2
C4H	20231211018 T	BS-E18068O2
PPO	20231211020 T	BS-E18012O2
ALDH	20231211021 T	BS-E18697O2
HCT	20231211019 T	BS-E19353O2

## Data Availability

The raw RNA-seq datasets can found in the NCBI SRA under the project number: PRJNA1180130. Available online: https://dataview.ncbi.nlm.nih.gov/object/PRJNA1180130?reviewer=2ine9doo881u1ib5i81bq0lsmn accessed on 30 December 2024.

## Supplementary Materials

The following supporting information can be downloaded at: https://www.mdpi.com/article/10.3390/ijms26020668/s1.

## Abbreviations

ANR: anthocyanidin reductase; ANS, anthocyamin synthase; C4H, cinnamate 4-hydroxylase; CHI, chalcone-flavanone isomerase; CHS, chalcone synthase; COMT, catechol-*O*-methyltransferase; 4CL, 4-coumarate-CoA ligase; DFR, dihydroflavonol-4-reductase; F3′H, flavonoid-3-hydroxylase; HCT, hydroxycinnamoyltransferase; LAR, leucoanthocyanidin reductase; LDOX, leucoanthocyanidin dioxygenase; PAL, phenylalanine ammonia-lyase; PPO, polyphenol oxidase; UFGT, UDP-flavonoid glucosyltransferase; UGT, UDP-glucosyltransferase; URT, rhamnosyltransferase.

## References

[1] Nq D., Hx L. (2021). Contribution of Phenylpropanoid Metabolism to Plant Development and Plant-Environment Interactions. J. Integr. Plant Biol..

[2] Liu Y., Liu J., Tang C., Uyanga V.A., Xu L., Zhang F., Sun J., Chen Y. (2024). Flavonoids-targeted Metabolomic Analysis Following Rice Yellowing. Food Chem..

[3] Crizel R.L., Perin E.C., Siebeneichler T.J., Borowski J.M., Messias R.S., Rombaldi C.V., Galli V. (2020). Abscisic Acid and Stress Induced by Salt: Effect on the Phenylpropanoid, L-Ascorbic Acid and Abscisic Acid Metabolism of Strawberry Fruits. Plant Physiol. Biochem..

[4] Sheng X., Chen H., Wang J., Zheng Y., Li Y., Jin Z., Li J. (2021). Joint Transcriptomic and Metabolic Analysis of Flavonoids in *Cyclocarya paliurus* Leaves. ACS Omega.

[5] Liu W., Feng Y., Yu S., Fan Z., Li X., Li J., Yin H. (2021). The Flavonoid Biosynthesis Network in Plants. Int. J. Mol. Sci..

[6] Cheng N., Wang H., Hao H., Rahman F.U., Zhang Y. (2023). Research Progress on Polysaccharide Components of *Cistanche deserticola* as Potential Pharmaceutical Agents. Eur. J. Med. Chem..

[7] Wu L., Georgiev M., Cao H., Nahar L., El-Seedi H., Sarker S., Xiao J., Lu B. (2020). Therapeutic potential of phenylethanoid glycosides: A systematic review. Med. Res. Rev..

[8] Tian X., Li M., Lin T., Qiu Y., Zhu Y., Li X., Tao W., Wang P., Ren X., Chen L. (2021). A review on the structure and pharmacological activity of phenylethanoid glycosides. Eur. J. Med. Chem..

[9] Wang X., Wang J., Guan H., Xu R., Luo X., Su M., Chang X., Tan W., Chen J., Shi Y. (2017). Comparison of the Chemical Profiles and Antioxidant Activities of Different Parts of Cultivated *Cistanche deserticola* Using Ultra Performance Liquid Chromatography-Quadrupole Time-of-Flight Mass Spectrometry and a 1,1-Diphenyl-2-picrylhydrazyl-Based Assay. Molecules.

[10] Xue Z., Yang B. (2016). Phenylethanoid Glycosides: Research Advances in Their Phytochemistry, Pharmacological Activity and Pharmacokinetics. Molecules.

[11] Alipieva K., Korkina L., Orhan I., Georgiev M. (2014). Verbascoside--a review of its occurrence, (bio)synthesis and pharmacological significance. Biotechnol. Adv..

[12] Zhang M., Ren X., Yue S., Zhao Q., Shao C., Wang C. (2019). Simultaneous Quantification of Four Phenylethanoid Glycosides in Rat Plasma by UPLC-MS/MS and Its Application to a Pharmacokinetic Study of *Acanthus ilicifolius* Herb. Molecules.

[13] Zhou J., Zhang Q., Sun J., Wang F., Zeng P. (2014). Simultaneous separation and determination of four phenylethanoid glycosides in rat plasma sample after oral administration of *Cistanche salsa* extract by microemulsion liquid chromatography. J. Chromatogr. B Analyt Technol. Biomed. Life Sci..

[14] Yan Y., Mo T., Huang W., Xu X., Tian W., Wang Y., Song Y., Li J., Shi S., Liu X. (2022). Glycosylation of Aromatic Glycosides by a Promiscuous Glycosyltransferase UGT71BD1 from *Cistanche tubulosa*. J. Nat. Prod..

[15] Lei L., Song Z.H., Tu P., Wu L., Chen F. (2001). Separation of echinacoside by reversed-phase preparative high performance liquid chromatography. Se Pu.

[16] Qiao F., Lu Y., Geng G., Zhou L., Chen Z., Wang L., Xie H., Qiu Q.-S. (2023). Flavonoid Synthesis in *Lamiophlomis rotata* from Qinghai-Tibet Plateau Is Influenced by Soil Properties, Microbial Community, and Gene Expression. J. Plant Physiol..

[17] Li Y., Li F., Zheng T.-T., Shi L., Zhang Z.-G., Niu T.-M., Wang Q.-Y., Zhao D.-S., Li W., Zhao P. (2021). Lamiophlomis Herba: A Comprehensive Overview of Its Chemical Constituents, Pharmacology, Clinical Applications, and Quality Control. Biomed. Pharmacother..

[18] Huang X.-J., Wang J., Muhammad A., Tong H.-Y., Wang D.-G., Li J., Ihsan A., Yang G.-Z. (2021). Systems Pharmacology-Based Dissection of Mechanisms of Tibetan Medicinal Compound Ruteng as an Effective Treatment for Collagen-Induced Arthritis Rats. J. Ethnopharmacol..

[19] Cui Z.-H., Qin S.-S., Zang E.-H., Li C., Gao L., Li Q.-C., Wang Y.-L., Huang X.-Z., Zhang Z.-Y., Li M.-H. (2020). Traditional Uses, Phytochemistry, Pharmacology and Toxicology of *Lamiophlomis rotata* (Benth.) Kudo: A Review. RSC Adv..

[20] Zhang S., Wang X., Yang J., Lu S., Lai X.-H., Jin D., Huang Y., Zhu W., Li J., Pu J. (2020). Nocardioides Dongxiaopingii Sp. Nov., Isolated from Leaves of *Lamiophlomis rotata* on the Qinghai-Tibet Plateau. Int. J. Syst. Evol. Microbiol..

[21] Song Y., Liang Y., Zeng R., Li R., Zhou Y., Huang S., Li X., Zhang N., Xu M., Xiong K. (2023). Qualitative and Quantitative Analyses of Chemical Constituents in Vitro and in Vivo and Systematic Evaluation of the Pharmacological Effects of Tibetan Medicine Zhixue Zhentong Capsules. Front. Pharmacol..

[22] Lian C., Zhang B., Yang J., Lan J., Yang H., Guo K., Li J., Chen S. (2022). Validation of Suitable Reference Genes by Various Algorithms for Gene Expression Analysis in *Isodon rubescens* under Different Abiotic Stresses. Sci. Rep..

[23] Zhou Z., Li T., Du R., Liu C., Huang S., Han L., Zhang P., Wang Y., Jiang M. (2023). *Lamiophlomis rotata* Attenuates Rheumatoid Arthritis by Regulating Sphingolipid and Steroid Hormone Metabolism. Mol. Omics.

[24] Zhan H., Chen R., Zhong M., Wang G., Jiang G., Tao X., Chen M., Jiang Y. (2023). Exploring the Pharmacological Mechanisms and Key Active Ingredients of Total Flavonoids from *Lamiophlomis rotata* (Benth.) Kudo against Rheumatoid Arthritis Based on Multi-Technology Integrated Network Pharmacology. J. Ethnopharmacol..

[25] Zhang D., Gao Y.-L., Jiang S., Chen Y., Zhang Y., Pan Z. (2018). The Similarity and Variability of the Iridoid Glycoside Profile and Antioxidant Capacity of Aerial and Underground Parts of *Lamiophlomis rotata* According to UPLC-TOF-MS and Multivariate Analyses. RSC Adv..

[26] Peng P., Zou J., Zhong B., Zhang G., Zou X., Xie T. (2023). Protective Effects and Mechanisms of Flavonoids in Renal Ischemia-Reperfusion Injury. Pharmacology.

[27] Majee C., Mazumder R., Choudhary A.N., Salahuddin (2023). An Insight into the Hepatoprotective Activity and Structure-Activity Relationships of Flavonoids. Mini-Rev. Med. Chem..

[28] Kumar G.A., Kumar S., Bhardwaj R., Swapnil P., Meena M., Seth C.S., Yadav A. (2023). Recent Advancements in Multifaceted Roles of Flavonoids in Plant-Rhizomicrobiome Interactions. Front. Plant Sci..

[29] Wang Y., Li T., Weng X. (2024). Response Letter to Hung-Hsuan Wang et al. on Efficacy and Safety of Gutong Patch Compared with NSAIDs for Knee Osteoarthritis: A Real-World Multicenter, Prospective Cohort Study in China. Pharmacol. Res..

[30] Ghosh D., Konishi T. (2007). Anthocyanins and Anthocyanin-Rich Extracts: Role in Diabetes and Eye Function. Asia Pac. J. Clin. Nutr..

[31] Muruganathan N., Dhanapal A.R., Baskar V., Muthuramalingam P., Selvaraj D., Aara H., Shiek Abdullah M.Z., Sivanesan I. (2022). Recent Updates on Source, Biosynthesis, and Therapeutic Potential of Natural Flavonoid Luteolin: A Review. Metabolites.

[32] Xu H., Yu S., Lin C., Dong D., Xiao J., Ye Y., Wang M. (2024). Roles of Flavonoids in Ischemic Heart Disease: Cardioprotective Effects and Mechanisms against Myocardial Ischemia and Reperfusion Injury. Phytomed. Int. J. Phytother. Phytopharm..

[33] Calderaro A., Patanè G.T., Tellone E., Barreca D., Ficarra S., Misiti F., Laganà G. (2022). The Neuroprotective Potentiality of Flavonoids on Alzheimer’s Disease. Int. J. Mol. Sci..

[34] Yue H.-L., Zhao X.-H., Mei L.-J., Shao Y. (2013). Separation and Purification of Five Phenylpropanoid Glycosides from *Lamiophlomis rotata* (Benth.) Kudo by a Macroporous Resin Column Combined with High-Speed Counter-Current Chromatography. J. Sep. Sci..

[35] Xia M., Zhang Y., Wu H., Zhang Q., Liu Q., Li G., Zhao T., Liu X., Zheng S., Qian Z. (2022). Forsythoside B Attenuates Neuro-Inflammation and Neuronal Apoptosis by Inhibition of NF-κB and P38-MAPK Signaling Pathways through Activating Nrf2 Post Spinal Cord Injury. Int. Immunopharmacol..

[36] Wang L., Geng G., Xie H., Zhou L., He Y., Li Z., Qiao F. (2024). A Transcriptomic and Metabolomic Study on the Biosynthesis of Iridoids in *Phlomoides rotata* from the Qinghai-Tibet Plateau. Plants.

[37] Lei L., Wan G., Geng X., Sun J., Zhang Y., Wang J., Yang C., Pan Z. (2023). The Total Iridoid Glycoside Extract of *Lamiophlomis rotata* Kudo Induces M2 Macrophage Polarization to Accelerate Wound Healing by RAS/ P38 MAPK/NF-κB Pathway. J. Ethnopharmacol..

[38] Li Z., Geng G., Xie H., Zhou L., Wang L., Qiao F. (2024). Metabolomic and Transcriptomic Reveal Flavonoid Biosynthesis and Regulation Mechanism in *Phlomoides rotata* from Different Habitats. Genomics.

[39] Wu R., Qian C., Yang Y., Liu Y., Xu L., Zhang W., Ou J. (2024). Integrative Transcriptomic and Metabolomic Analyses Reveal the Phenylpropanoid and Flavonoid Biosynthesis of *Prunus mume*. J. Plant Res..

[40] Lv H., Guo S. (2023). Comparative Analysis of Flavonoid Metabolites from Different Parts of *Hemerocallis citrina*. BMC Plant Biol..

[41] Shi M., Ali M., He Y., Ma S., Rizwan H., Yang Q., Li B., Lin Z., Chen F. (2021). Flavonoids Accumulation in Fruit Peel and Expression Profiling of Related Genes in Purple (*Passiflora edulis* f. *edulis*) and Yellow (*Passiflora edulis* f. *flavicarpa*) Passion Fruits. Plants.

[42] Liu L., Zhang Y., Jiang X., Du B., Wang Q., Ma Y., Liu M., Mao Y., Yang J., Li F. (2023). Uncovering nutritional metabolites and candidate genes involved in flavonoid metabolism in *Houttuynia cordata* through combined metabolomic and transcriptomic analyses. Plant Physiol. Biochem..

[43] Yu Z.-W., Zhang N., Jiang C.-Y., Wu S.-X., Feng X.-Y., Feng X.-Y. (2021). Exploring the Genes Involved in Biosynthesis of Dihydroquercetin and Dihydromyricetin in *Ampelopsis grossedentata*. Sci. Rep..

[44] Waki T., Takahashi S., Nakayama T. (2021). Managing Enzyme Promiscuity in Plant Specialized Metabolism: A Lesson from Flavonoid Biosynthesis: Mission of a “Body Double” Protein Clarified. Bioessays.

[45] Zhu X., Mi Y., Meng X., Zhang Y., Chen W., Cao X., Wan H., Yang W., Li J., Wang S. (2022). Genome-Wide Identification of Key Enzyme-Encoding Genes and the Catalytic Roles of Two 2-Oxoglutarate-Dependent Dioxygenase Involved in Flavonoid Biosynthesis in *Cannabis sativa* L.. Microb. Cell Fact..

[46] Peng W., Wang N., Wang S., Wang J., Bian Z. (2023). Effect of Co-Treatment of Microwave and Exogenous l-Phenylalanine on the Enrichment of Flavonoids in *Tartary buckwheat* Sprouts. J. Sci. Food Agric..

[47] Tegl G., Nidetzky B. (2020). Leloir Glycosyltransferases of Natural Product C-Glycosylation: Structure, Mechanism and Specificity. Biochem. Soc. Trans..

[48] Li J., Liu X., Gao Y., Zong G., Wang D., Liu M., Fei S., Wei Y., Yin Z., Chen J. (2019). Identification of a UDP-Glucosyltransferase Favouring Substrate- and Regio-Specific Biosynthesis of Flavonoid Glucosides in *Cyclocarya paliurus*. Phytochemistry.

[49] Pei T., Yan M., Li T., Li X., Yin Y., Cui M., Fang Y., Liu J., Kong Y., Xu P. (2022). Characterization of UD-glycosyltransferase family members reveals how major flavonoid glycoside accumulates in the roots of *Scutellaria baicalensis*. BMC Genom..

[50] Du Z., Lin W., Yu B., Zhu J., Li J. (2021). Integrated Metabolomic and Transcriptomic Analysis of the Flavonoid Accumulation in the Leaves of *Cyclocarya paliurus* at Different Altitudes. Front. Plant Sci..

[51] Dong Q.-J., Xu X.-Y., Fan C.-X., Xiao J.-P. (2024). Transcriptome and Metabolome Analyses Reveal Chlorogenic Acid Accumulation in *Pigmented* Potatoes at Different Altitudes. Genomics.

[52] Suzuki A., Silsirivanit A., Watanabe T., Matsuda J., Inamori K.I., Inokuchi J.I. (2023). Mass Spectrometry of Neutral Glycosphingolipids. Methods Mol. Biol..

[53] Liu Y., Li Y., Liu Z., Wang L., Bi Z., Sun C., Yao P., Zhang J., Bai J., Zeng Y. (2023). Integrated Transcriptomic and Metabolomic Analysis Revealed Altitude-Related Regulatory Mechanisms on Flavonoid Accumulation in Potato Tubers. Food Res. Int..

[54] Grabherr M.G., Haas B.J., Yassour M., Levin J.Z., Thompson D.A., Amit I., Adiconis X., Fan L., Raychowdhury R., Zeng Q. (2011). Full-Length Transcriptome Assembly from RNA-Seq Data without a Reference Genome. Nat. Biotechnol..

[55] Buchfink B., Xie C., Huson D.H. (2015). Fast and Sensitive Protein Alignment Using DIAMOND. Nat. Methods.

[56] Tang L., Liu J., Liu L., Yu Y., Zhao H., Lu W. (2020). De Novo Transcriptome Identifies Olfactory Genes in *Diachasmimorpha longicaudata* (Ashmead). Genes.

[57] Yan W.-J., Hussain H., Chung H.H., Julaihi N., Tommy R. (2022). Transcriptome Dataset of *Sago palm* in Peat Soil. Data Brief..

[58] Tatusov R.L. (2000). The COG Database: A Tool for Genome-Scale Analysis of Protein Functions and Evolution. Nucleic Acids Res..

[59] Koonin E.V., Fedorova N.D., Jackson J.D., Jacobs A.R., Krylov D.M., Makarova K.S., Mazumder R., Mekhedov S.L., Nikolskaya A.N., Rao B.S. (2004). A Comprehensive Evolutionary Classification of Proteins Encoded in Complete Eukaryotic Genomes. Genome Biol..

[60] Huerta-Cepas J., Szklarczyk D., Forslund K., Cook H., Heller D., Walter M.C., Rattei T., Mende D.R., Sunagawa S., Kuhn M. (2016). eggNOG 4.5: A Hierarchical Orthology Framework with Improved Functional Annotations for Eukaryotic, Prokaryotic and Viral Sequences. Nucleic Acids Res..

[61] Kanehisa M. (2004). The KEGG Resource for Deciphering the Genome. Nucleic Acids Res..

[62] Love M.I., Huber W., Anders S. (2014). Moderated Estimation of Fold Change and Dispersion for RNA-Seq Data with DESeq2. Genome Biol..

[63] Xie C., Mao X., Huang J., Ding Y., Wu J., Dong S., Kong L., Gao G., Li C.-Y., Wei L. (2011). KOBAS 2.0: A Web Server for Annotation and Identification of Enriched Pathways and Diseases. Nucleic Acids Res..

[64] Livak K., Schmittgen T. (2001). Analysis of Relative Gene Expression Data Using Real-Time Quantitative PCR and the 2^−ΔΔCt^ Method. Methods.

