# Supplementary material for: Integrated Metabolome, Transcriptome, and Physiological Analysis of the Flavonoid and Phenylethanol Glycosides Accumulation in Wild Phlomoides rotata Roots from Different Habitats
Source: Int J Mol Sci. 2025 Jan 14;26(2):668. doi: 10.3390/ijms26020668 (PMC11766294; doi:10.3390/ijms26020668)
Supplement: Supplementary file 1 [file ijms-26-00668-s001.zip › Tables .pdf]

Table S2 Flavonoids and PhGs metabolite classification in *P. rotata* roots from 4 habitats

ID	name	Class I	Class II	Number
POS_q71	Cyanidin-3-O-Galactoside (Chloride)	Flavonoids	Polyphenols	1
POS_q99	Fisetin	Flavonoids	Polyphenols	2
POS_q117	Irisolidone	Flavonoids	Polyphenols	3
POS_q147	Luteolin 7-Diglucuronide	Flavonoids	Polyphenols	4
POS_q150	Marein	Flavonoids	Polyphenols	5
POS_q191	Rutin	Flavonoids	Polyphenols	6
NEG_t52	Glabrene	Flavonoids	Other Flavonoids	7
NEG_q228	Safflower Yellow	Flavonoids	Other	8
POS_q70	Cyanidin-3-O-Arabinoside Chloride	Flavonoids	Other	9
POS_q149	Malvidin Chloride	Flavonoids	Other	10
POS_q31	7-Hydroxy-4H-Chromen-4-One	Flavonoids	Monophenols	11
POS_q217	Wogonoside	Flavonoids	Monophenols	12
NEG_q66	Biochanin A	Flavonoids	Isoflavones	13
POS_q100	Formononetin	Flavonoids	Isoflavones	14
POS_q111	Homoferreirin	Flavonoids	Isoflavones	15
NEG_q148	Isoswertisin 2''-Rhamnoside	Flavonoids	Hydroxyflavone	16
POS_q40	Apiin	Flavonoids	Hydroxyflavone	17
POS_q53	Breviscapin	Flavonoids	Hydroxyflavone	18
NEG_q229	Sakuranetin	Flavonoids	Flavonones	19
NEG_q157	Leucoside	Flavonoids	Flavonols	20
NEG_q191	Narcissin	Flavonoids	Flavonols	21
POS_q63	Chrysosplenol D	Flavonoids	Flavonols	22
POS_q113	Ikarisoside A	Flavonoids	Flavonols	23
POS_q124	Kaempferol 3-Neohesperidoside	Flavonoids	Flavonols	24
POS_q166	Nicotiflorin	Flavonoids	Flavonols	25
POS_q188	Rhamnocitrin 3-Glucoside	Flavonoids	Flavonols	26
POS_q6	2,3-Dehydrosilybin A	Flavonoids	Flavonolignans	27
POS_q82	Dichotomitin	Flavonoids	Flavonoids	28
NEG_q203	Oroxylin A-7-O-Glucuronide	Flavonoids	Flavones	29
NEG_q264	Vicenin 2	Flavonoids	Flavones	30
POS_q23	5,7-Dimethoxyflavone	Flavonoids	Flavones	31
POS_q38	Albanin A	Flavonoids	Flavones	32
POS_q142	Lonicerin	Flavonoids	Flavones	33
POS_q152	Meloside A	Flavonoids	Flavones	34
POS_q216	Vitexin	Flavonoids	Flavones	35
NEG_t46	Acacetin-7-O-rutinoside (Linarin)	Flavonoids	Flavones	36
POS_t54	3-Hydroxyphloretin	Flavonoids	Chalcones	37
POS_q69	Cupressuflavone	Flavonoids	Biflavones	38
NEG_q7	1-Phenylethanol	Phenylpropanoids	Simple Phenylpropanols	39
NEG_q20	3,4-Dimethoxycinnamic Acid	Phenylpropanoids	Simple Phenylpropanols	40
NEG_q32	5-Methoxy-7-Hydroxycoumarin	Phenylpropanoids	Simple Phenylpropanols	41
NEG_q67	Calceolarioside B	Phenylpropanoids	Simple Phenylpropanols	42
NEG_q71	Cichoriin	Polyphenols	Polyphenols	43
NEG_q82	Demethylwedelolactone	Sugars and alcohols	Other	44

NEG_q98	Dl-Tyrosine	Coumarins	Other	45
NEG_q118	Forsythiaside A	Amino acids	Other	46
NEG_q119	Forsythoside B	Polyphenols	Other	47
NEG_q135	Hydroxytyrosol	Amino acids	Other	48
NEG_q213	Phenylpyruvic Acid	Amino acids	Other	49
NEG_q231	Salidroside	Xanthones	Other	50
NEG_q262	Verbascoside	Coumarins	Other	51
POS_q15	3-(3,4-Dihydroxy-5-Methoxy)-2-Propenoic Acid	Amino Acids	Other	52
POS_q18	3-Nitro-L-Tyrosine	Coumarins	Hydroxycoumarins	53
NEG_q166	L-Tyrosine	Amino Acids	Other	54
POS_q19	3-O-Methyldopa	Polyphenols	Glycosides	55
POS_q119	Isogentisin	Coumarins	Coumarins	56
POS_q148	Luvangetin-1	Others	Cinnamic Acids And Derivatives	57
POS_q173	N-Trans-Sinapoyltyramine	Organic acid	Cinnamic Acids And Derivatives	58
POS_t68	Phe-His	Organic acid	Carboxylic Acids	59

Note: yellow background covered flavonoids (38), and no background covered phenylethanol glycosides (21).

Table S3 Transcriptome quality control

ID	Read number	Base number	GC Content	≥Q30
HN1	20,767,307	6,215,771,45	45.55%	95.17%
HN2	19,641,152	5,876,870,704	45.52%	95.19%
HN3	20,660,140	6,182,997,472	45.50%	94.73%
GL1	21,966,204	6,565,961,114	45.98%	93.89%
GL2	21,120,829	6,312,156,194	44.55%	94.80%
GL3	21,500,946	6,428,942,196	45.06%	94.45%
YS1	19,893,404	5,948,445,960	45.80%	95.61%
YS2	20,180,763	6,034,840,972	44.96%	94.86%
YS3	20,375,010	6,095,830,148	44.82%	94.66%
CD1	20,836,693	6,228,842,428	45.57%	94.84%
CD2	19,943,688	5,960,640,924	46.03%	95.29%
CD3	20,041,886	5,992,432,296	45.74%	95.35%

Table S4 Unigene Annotation Statistics Table

#Anno_Database	Annotated_Number	300<=length<1000	length>=1000
COG_Annotation	7,099	1,988	5,111
GO_Annotation	18,406	6,072	12,329
KEGG_Annotation	15,207	4,706	10,501
KOG_Annotation	12,901	4,007	8,894
Pfam_Annotation	17,074	4,931	12,143
Swissprot_Annotation	14,647	4,270	10,377
TrEMBL_Annotation	21,454	7,392	14,062

eggNOG_Annotation	17,911	5,675	12,236
nr_Annotation	23,041	8,416	14,625
All_Annotated	23,540	8,812	14,723

Table S5 Genes in the flavonoid pathway of *P. rotata* roots.

Number	Gene name	NCBI Transcript number
1	*4CL1*	TRINITY_DN2078_c0_g2
2	*4CL2*	TRINITY_DN43283_c0_g1
3	*4CL3*	TRINITY_DN555_c0_g3
4	*CHS1*	TRINITY_DN15801_c0_g1
5	*CHS2*	TRINITY_DN1746_c1_g1
6	*CHI1*	TRINITY_DN14941_c0_g1
7	*CHI2*	TRINITY_DN15801_c0_g1
8	*F3'H1*	TRINITY_DN10722_c0_g3
9	*DFR1*	TRINITY_DN1110_c1_g1
10	*UFGT1*	TRINITY_DN8617_c0_g1
11	*UFGT2*	TRINITY_DN4249_c0_g1
12	*UFGT3*	TRINITY_DN42_c0_g1
13	*UFGT4*	TRINITY_DN1208_c1_g1
14	*UFGT5*	TRINITY_DN13761_c0_g1
15	*UFGT6*	TRINITY_DN6291_c0_g1
16	*UFGT7*	TRINITY_DN6291_c0_g1
17	*UFGT8*	TRINITY_DN225894_c0_g1
18	*UFGT9*	TRINITY_DN31608_c0_g2
19	*COMT1*	TRINITY_DN6672_c1_g1
20	*COMT2*	TRINITY_DN10323_c0_g1
21	*ANR1*	TRINITY_DN2413_c0_g1
22	*HCT1*	TRINITY_DN10722_c0_g2
23	*HCT2*	TRINITY_DN10722_c0_g3
24	*HCT3*	TRINITY_DN4065_c0_g1

Table S6 Genes in PhGs pathway of *P. rotata* roots.

Number	Gene name	NCBI Transcript number
1	*PAL1*	TRINITY_DN2246_c0_g1
2	*PAL2*	TRINITY_DN2246_c1_g1
3	*4CL1*	TRINITY_DN2078_c0_g2

4	*4CL2*	TRINITY_DN43283_c0_g1
5	*4CL3*	TRINITY_DN555_c0_g3
6	*ALDH1*	TRINITY_DN11749_c0_g1
7	*ALDH2*	TRINITY_DN2565_c0_g1
8	*ALDH3*	TRINITY_DN3427_c0_g1
9	*C4H1*	TRINITY_DN4065_c0_g1
10	*C4H2*	TRINITY_DN3113_c1_g1
11	*C4H3*	TRINITY_DN212_c0_g1
12	*C4H4*	TRINITY_DN1245_c0_g1
13	*C4H5*	TRINITY_DN212_c0_g1
14	*C4H6*	TRINITY_DN1245_c0_g1
15	*C4H7*	TRINITY_DN4065_c0_g1
16	*C4H8*	TRINITY_DN3113_c1_g1
17	*URT*	TRINITY_DN4249_c0_g2
18	*HCT1*	TRINITY_DN6291_c0_g1
19	*HCT2*	TRINITY_DN950_c0_g1
20	*HCT3*	TRINITY_DN4065_c0_g1
21	*UGT1*	TRINITY_DN2720_c0_g1
22	*UGT2*	TRINITY_DN4249_c0_g2
23	*UGT3*	TRINITY_DN6291_c0_g1
24	*UGT4*	TRINITY_DN950_c0_g1
25	*PPO1*	TRINITY_DN219_c1_g2
26	*PPO2*	TRINITY_DN219_c2_g1

Table S8 Mass spectrometry conditions.

Mass spectrometry conditions.	Parameter
Temperature of electric spray ion source	550°C
Ion Spray Voltage	5500 V/ - 4500 V
GSI, GSII, and curtain air	50, 55, and 35 psi
Collision induced ionization parameters	secondary
Collision gas (nitrogen)	secondary
Cluster voltage and collision energy	Specific optimization

Table S9 qRT-PCR primers and sequences of related genes in the flavonoid and PhGs pathway of *P. rotata* roots.

Number	Gene name	NCBI Transcript number	Primer sequence	Length
1	*PAL1*	TRINITY_DN2246_c0_g1	F: AGCACCATTCCAAGCCTCAAGAC<br>R: AGGGAGGAAGTTGGGACAGAGTA	141
2	*PAL2*	TRINITY_DN2246_c1_g1	F: CAGATTCTCCTCCAAATGCCTCA<br>R: AGCAGCATAACCAGGATGTGAACT	146
3	*4CL1*	TRINITY_DN2078_c0_g2	F: CCGGTGCTCGCTTTCACTATCCC<br>R: TCTCATCATTTCCTCTATCGTCGTA	135
4	*4CL2*	TRINITY_DN43283_c0_g1	F: CCTGTCGTCGGTGAGGAAAGTGA<br>R: TGCTTCCGTCATACCATAACCCT	121
5	*4CL3*	TRINITY_DN555_c0_g3	F: CATCCCGAAACACCTTCCATTAC<br>R: GCGTTGCTCCGTCCATTAGACAT	89
6	*4CL4*	c679040.graph_c0	F: GGGTGGTTGAGAACTGGAGAT<br>R: GGAGGAACCTGATAACCCTTG	107
7	*CHS1*	TRINITY_DN15801_c0_g1	F: CAAAGAAGCTGCCCTGAAAG<br>R: GGTGGTGGTGCAGAATATGAG	82
8	*CHS2*	TRINITY_DN1746_c1_g1	F: CGAGAAACTAAGGTCGACACG<br>R: CGGAGGACTTCCTTCATCTCAT	98
9	*CHI1*	TRINITY_DN14941_c0_g1	F: CCCATCTCCAGAAATGGAAGG<br>R: CCAACTTTTCCACAGGAGCAG	96
10	*CHI2*	TRINITY_DN15801_c0_g1	F: GGGGCGAGAGGCTTAGAGAT<br>R: CAATGGAACGGCGTTATCCT	84
11	*F3'H2*	TRINITY_DN10722_c0_g3	F: TGGGTTCAAGAGACCGACTTG<br>R: GCGCAATGAGGGGCTAATAA	104
12	*DFR1*	TRINITY_DN1110_c1_g1	F: CTACTGTAGGGGGTCCGTTCC<br>R: CTACTGTAGGGGGTCCGTTCC	92
13	*UFGT1*	TRINITY_DN8617_c0_g1	F: TAACTCACTGCGGGTGGAACT<br>R: GGAATCCCCGTCTTCAGAAT	130
14	*HCT1*	TRINITY_DN10722_c0_g2	F: GGTATCATCCGTCACTTTCTCCAC<br>R: AGCAAACCAGCCTCCACTTCGTC	188
15	*HCT2*	TRINITY_DN10722_c0_g3	F: TGTTTCATCCGTAACTTTCTCCACG<br>R: ACAGCCTACAAGCCTCCACTTCG	190
16	*HCT3*	TRINITY_DN7911_c0_g1	F: TGCCTTCTTCTTCTTCAGTAGTTGT<br>R: TTGGAGGGATTTGAGGGTGGATT	80
17	*UGT1*	TRINITY_DN2720_c0_g1	F: AAAGATGATTCGGACAAGGCTGAG<br>R: GCTTCCAAGGCAAACGTAGACGA	117
18	*UGT2*	TRINITY_DN4249_c0_g2	F: CATCCACAGTGCGTAACGAAACA<br>R: TGATCAGGCAGTCGAAGGAGAAA	139
19	*UGT3*	TRINITY_DN6291_c0_g1	F: GTTTCATCGTCCTCGTCTTCCTC<br>R: TTCGCTTGATGGTAGTGACATAGTG	147
20	*UGT4*	TRINITY_DN950_c0_g1	F: AAACTTCCCAACGAGACGTACAAA<br>R: AACCAAAGCGGCGATCATCACTA	83
21	*C4H1*	TRINITY_DN4065_c0_g1	F: CGGAATGTTTATCAGCAAAGGAC<br>R: GTGAGGCTCATATTGTTGACCAG	142
22	*C4H2*	TRINITY_DN3113_c1_g1	F: TACGAGCCTCACTATGATTACTT<br>R: AAAATTGTCTCACCACCTTCTTC	116
23	*C4H3*	TRINITY_DN212_c0_g1	F: AGGATTCTTCTCGCATTCGCCTTTA<br>R: TTGCACGGGAGCTGCCCTGTTAT	168
24	*C4H4*	TRINITY_DN1245_c0_g1	F: TTATAGTGCTGTGCCTTGGTGGA<br>R: AGTTGCGTCAGGTTTCATGCTCC	125

25	*C4H5*	TRINITY_DN3113_c1_g1	F: CAGAGCACGGAGAAGGTCTACAA<br>R: GTAGCTATGCGTTGACCTCCATT	118
26	*PPO1*	TRINITY_DN219_c1_g2	F: GAAGCGACGAGGAGAAGGAGAAG<br>R: TCGAACCTAACGGCATACGACAT	85
27	*PPO2*	TRINITY_DN219_c2_g1	F: CCCCTCACCCTGAAGAAGATTAC<br>R: CACGTCGAACTTGATGAACTTGGT	123
28	*ALDH1*	TRINITY_DN11749_c0_g1	F: TCCAATGCCACTCATCTTATACCC<br>R: CACTCATAACCTCGACACTGCTA	127

We have submitted the transcript sequences from *P.rotata* roots to NCBI. The raw RNA-seq datasets can found in the NCBI SRA under the project number: PRJNA1180130. https://dataview.ncbi.nlm.nih.gov/object/PRJNA1180130?reviewer=2ine9doo881u1ib5i81bq0lsmn.

Table S10 The reaction program of metabolites

Reaction program steps	temperature	time
1	95 ◦C	30 s,
2	95 ◦C	5 s
3	60 ◦C	30 s
4	40 cycles	
5	melting curve 95 ◦C	15 s
6	60 ◦C	50 s
7	95 ◦C	15 s
