# Supplementary figures and images for: Integrated Metabolome, Transcriptome, and Physiological Analysis of the Flavonoid and Phenylethanol Glycosides Accumulation in Wild Phlomoides rotata Roots from Different Habitats
Source: Int J Mol Sci. 2025 Jan 14;26(2):668. doi: 10.3390/ijms26020668 (PMC11766294; doi:10.3390/ijms26020668)

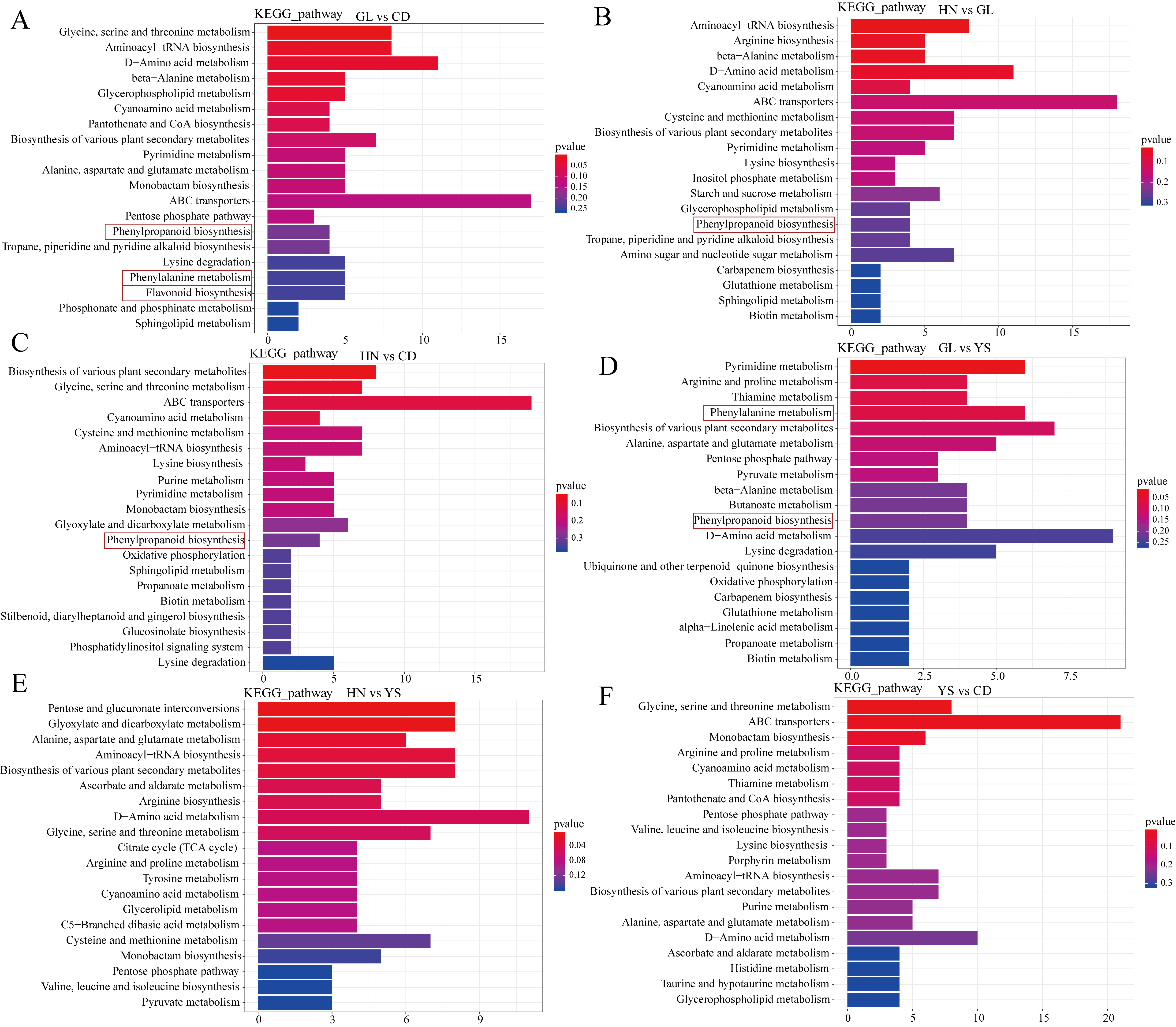

Supplement: Supplementary file 1 [file ijms-26-00668-s001.zip › Figure S1.tif]

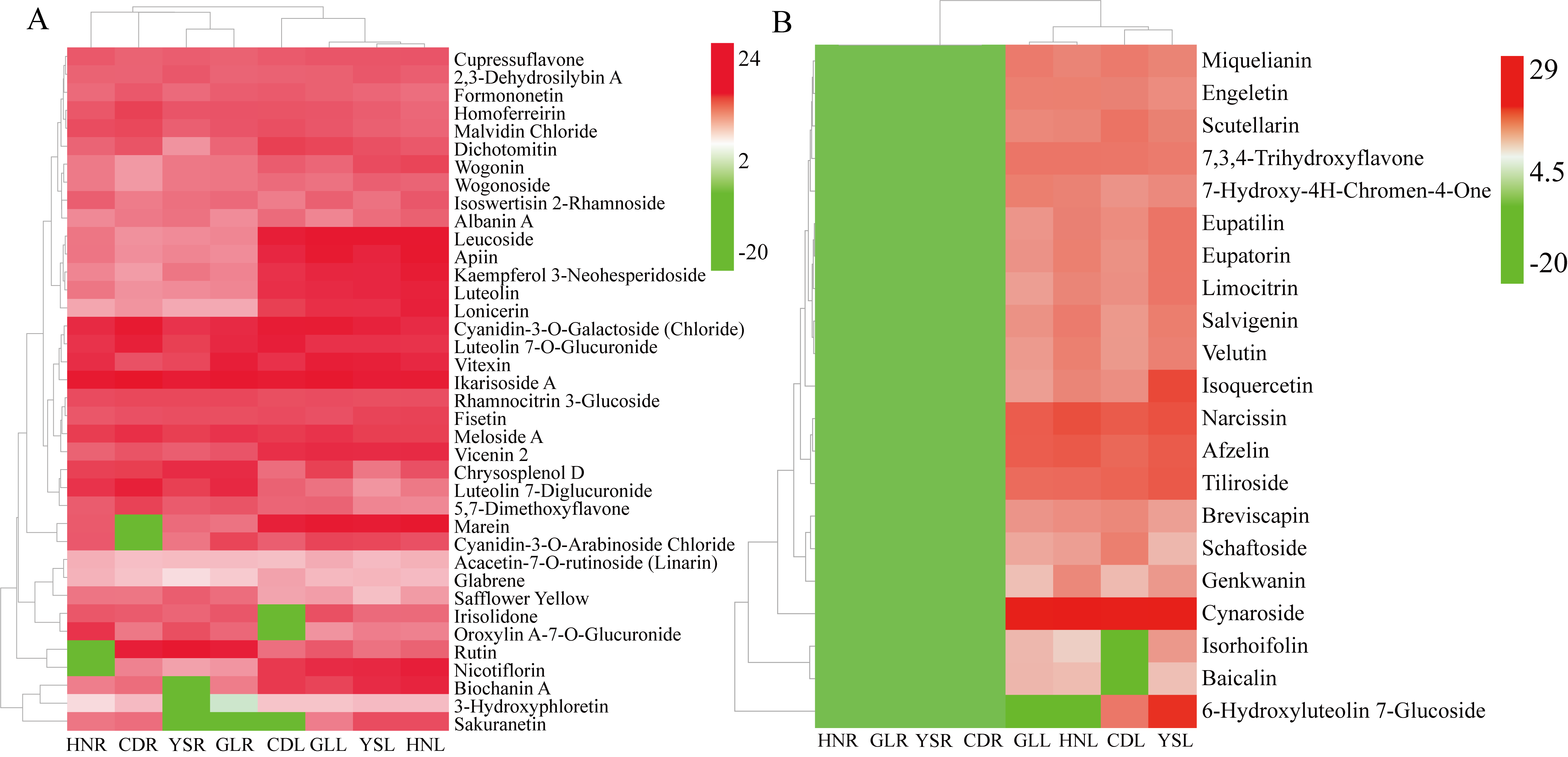

Supplement: Supplementary file 1 [file ijms-26-00668-s001.zip › Figure S3.tif]

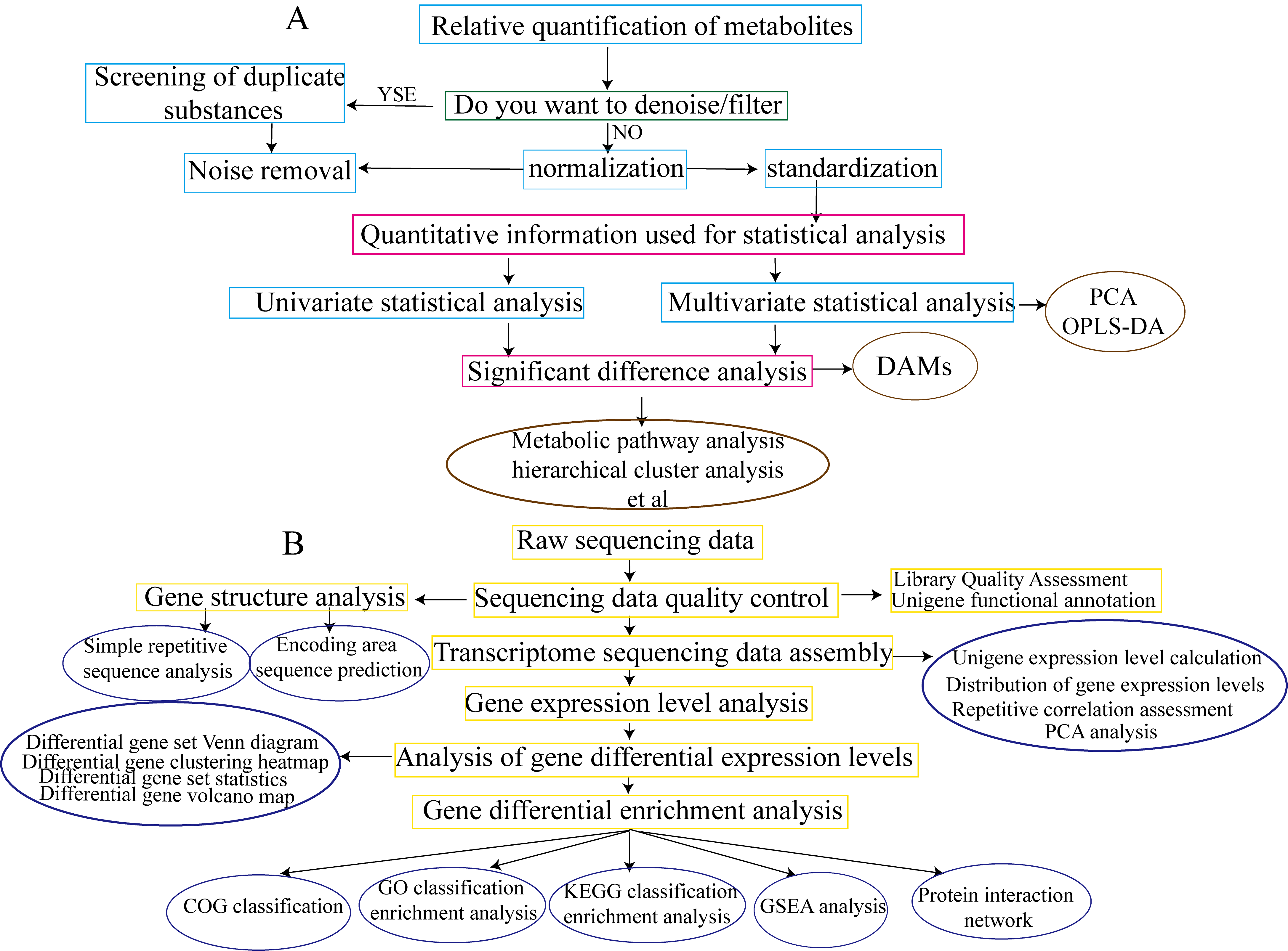

Supplement: Supplementary file 1 [file ijms-26-00668-s001.zip › Figure S4.tif]

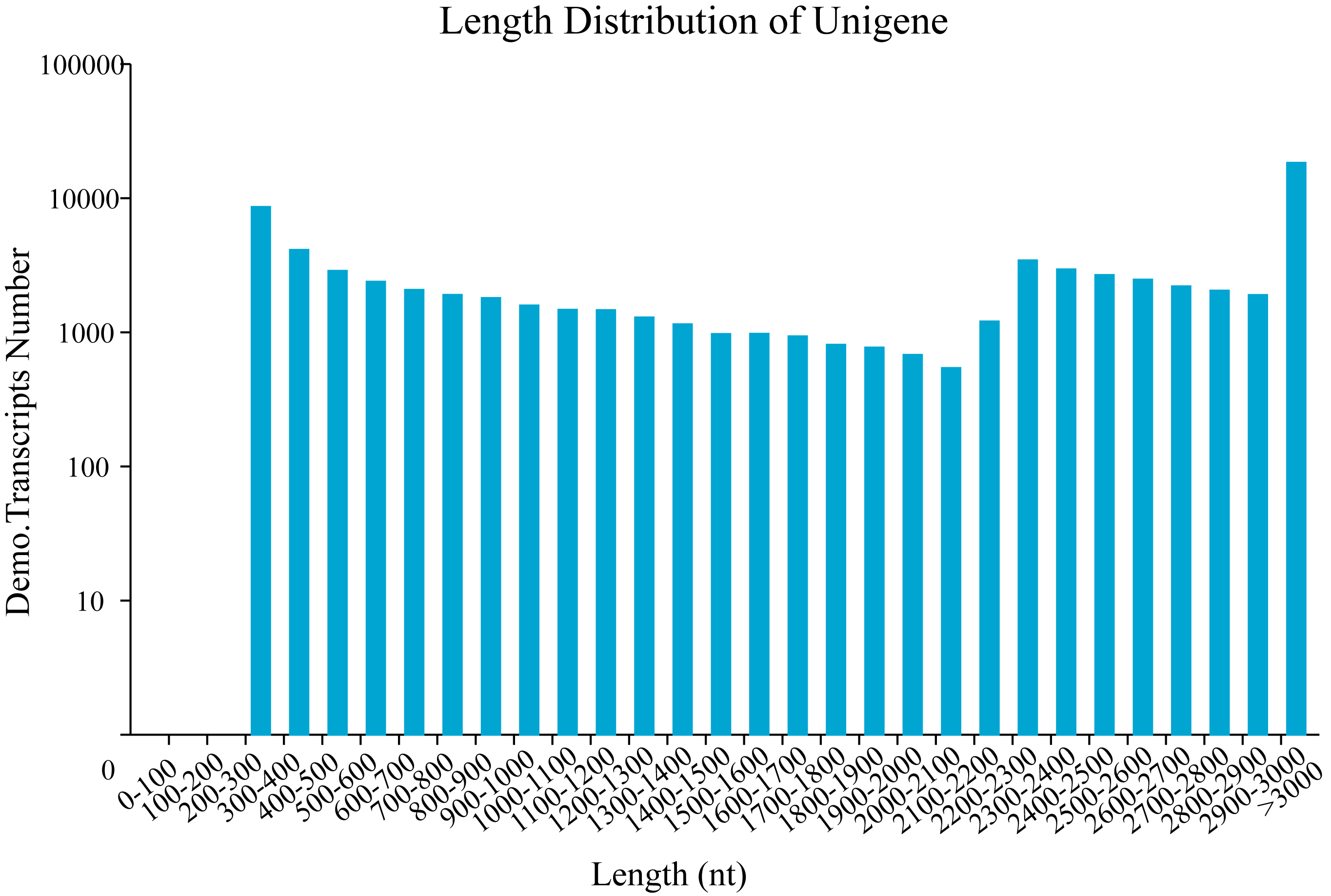

Supplement: Supplementary file 1 [file ijms-26-00668-s001.zip › FigureS2.tif]
